# Bioengineered Water-Responsive Carboxymethyl Cellulose/Poly(vinyl alcohol) Hydrogel Hybrids for Wound Dressing and Skin Tissue Engineering Applications

**DOI:** 10.3390/gels9020166

**Published:** 2023-02-18

**Authors:** Nádia Sueli Vieira Capanema, Alexandra Ancelmo Piscitelli Mansur, Isadora Cota Carvalho, Sandhra Maria Carvalho, Herman Sander Mansur

**Affiliations:** 1 Center of Nanoscience, Nanotechnology and Innovation—CeNano^2^I, Department of Metallurgical and Materials Engineering, Federal University of Minas Gerais, UFMG, Av. Presidente Antônio Carlos, 6627–Escola de Engenharia, Belo Horizonte 31270-901, MG, Brazil; 2 Departamento de Engenharia Agrícola, Universidade Federal de Lavras, UFLA, Lavras 37203-202, MG, Brazil; 3 Centro Universitário de Lavras, UNILAVRAS, Lavras 37203-593, MG, Brazil

**Keywords:** hydrogel, carboxymethyl cellulose, poly(vinyl alcohol), wound dressing, soft tissue engineering, polysaccharide-based hydrogels

## Abstract

The burden of chronic wounds is growing due to the increasing incidence of trauma, aging, and diabetes, resulting in therapeutic problems and increased medical costs. Thus, this study reports the synthesis and comprehensive characterization of water-responsive hybrid hydrogels based on carboxymethyl cellulose (CMC) and poly(vinyl alcohol) (PVA) using citric acid (CA) as the chemical crosslinking agent, with tunable physicochemical properties suitable to be applied as a wound dressing for soft tissue engineering applications. They were produced through an eco-friendly process under mild conditions. The hydrogels were designed and produced with flexible swelling degree properties through the selection of CMC molecular mass (Mw = 250 and 700 kDa) and degree of functionalization (DS = 0.81), degree of hydrolysis of PVA (DH > 99%, Mw = 84–150 kDa) associated with synthesis parameters, CMC/PVA ratio and extension of chemical crosslinking (CA/CMC:PVA ratio), for building engineered hybrid networks. The results demonstrated that highly absorbent hydrogels were produced with swelling degrees ranging from 100% to 5000%, and gel fraction from 40% to 80%, which significantly depended on the concentration of CA crosslinker and the presence of PVA as the CMC-based network modifier. The characterizations indicated that the crosslinking mechanism was mostly associated with the chemical reaction of CA carboxylic groups with hydroxyl groups of CMC and PVA polymers forming ester bonds, rendering a hybrid polymeric network. These hybrid hydrogels also presented hydrophilicity, permeability, and structural features dependent on the degree of crosslinking and composition. The hydrogels were cytocompatible with in vitro cell viability responses of over 90% towards model cell lines. Hence, it is envisioned that this research provides a simple strategy for producing biocompatible hydrogels with tailored properties as wound dressings for assisting chronic wound healing and skin tissue engineering applications.

## 1. Introduction

Skin is a very complex tissue and the largest organ of the body (approximately 15% of the body’s weight) that plays multiple critical roles, including a protective barrier against external factors, such as harmful materials (e.g., chemicals), radiation (e.g., ultraviolet), and pathogenic microbial agents. Moreover, it performs crucial functions in maintaining biological homeostasis, preventing the substantial loss of body fluids, assisting in thermoregulation, and acting as a defense for the body’s immune system. The skin is also a sensory organ that receives and transfers signals to the brain regarding the neighboring environment [1,2,3,4].

Since skin is directly in contact with the external environment, it always suffers from various physical or chemical damage, including burns, contusions, bruises, and other injuries and traumas, leading to the loss of its vital barrier functions. The disruption of the integrity or malfunction of the skin tissue is usually referred to as a wound [1,2,5]. So, when skin is injured or damaged, a typical process termed a *wound-healing cascade* is automatically triggered by the human body aiming to repair the wound sites [1,2]. Skin injuries often involve unwanted physical effects, such as pain, inflammation, mobility restriction, disturbance of sleep, and limitation of daily activities. Consequently, besides physical aspects, wounds harm patients’ quality of life, affecting their emotional and social states [1,2,3,4]. 

The repair of skin wounds is one of the most complex biological processes that occur during human life. Wound healing is a dynamic well-orchestrated process involving multiple interactions between cells, the extracellular matrix (ECM), and growth factors that reconstruct tissue following the injury [6,7,8]. Fortunately, healthy skin usually exhibits excellent regenerative properties, and normal skin wound healing involves a series of highly ordered processes. Typically, upon creating an injury or wound on the skin, the wound-healing process will undergo four major overlapping stages: hemostasis, inflammation, proliferation, and remodeling to heal. The normal wound-healing process usually occurs within 1 or 2 weeks [9,10,11]. However, due to several factors, some skin wounds are difficult to heal, including infections, excessive inflammatory reactions, localized or metabolic systemic diseases (e.g., diabetes), and substantial tissue loss, where the spontaneous wound-healing cascade fails to be implemented properly. Therefore, it causes imperfect skin repairs such as continuous inflammation, scar formation, and chronic wounds. Disrupting the carefully regulated normal healing process may hamper wound healing, creating chronic wounds such as venous or arterial ulcers, pressure sores, diabetic foot ulcers (DFU), and nonhealing surgical wounds [2,3,4,8,11,12]. Based on the healing process, skin wounds are usually classified into acute (“*normal*”) wounds and chronic wounds. The influence of single or multiple factors (e.g., diabetes, infection, inflammation, and reactive oxygen species (ROS)) at each stage of the healing process may delay its progression or even turn a *normal* wound into a chronic nonhealing wound [13,14]. Consequently, chronic skin wounds have become a major public health burden with an increasing frequency yearly, causing severe social and huge financial impacts globally [2,3,4,7,8,11].

Despite undeniable advances in regenerative medicine and tissue engineering, with a wide variety of artificial skin replacements commercially available for clinical use, skin repair and restoration remain challenging for the medical system. Numerous biomaterials have been designed and fabricated to assist with wound closure and the healing process. Still, no effective artificial skin that reproduces normal skin and restores all the damaged tissue functionalities has yet been developed. Hence, there is a growing demand for appropriate and safe biomaterials to treat and repair skin lesions and topical wounds that fulfill the injured tissue’s critical properties and functions [3,6,11,15,16]. 

Owing to providing a steady barrier and favorable microenvironment, the wound dressing is considered the most attractive strategy to promote wound healing. Ideally, an optimal wound dressing would provide a balanced moisture environment by minimizing dehydration and collecting exudates efficiently, avoiding wound contamination, being associated with an excellent in situ conformation, leading to expedited healing. Wound dressings comprise gauzes, electrospun scaffolds, foams and sponges, nanofibers, hydrocolloids, films, hydrogels, membranes, powders, and other structures similar to the extracellular matrix (ECM) [2,13,14,17]. Compared to other options for wound dressing, hydrogels have been increasingly accepted as an important solution for wound dressings to assist tissue repair because of their natural extracellular matrix (ECM)-mimicking structure with tunable properties [2,13,14,17]. Hydrogels are polymeric materials with three-dimensional (3D) network structures with hydrophilic macromolecular chains that can absorb and retain a large volume of water in their interstitial structures [6,18]. Hydrogels, with excellent wound exudate absorptive properties, hydrophilicity and moisture-retention capacity, and oxygen permeability have been widely accepted as the most promising wound dressings with shape adaptability to cover irregular wounds. Hydrogel-based wound dressings possess a unique hydrophilic network with high water retention, creating a moist and permeable microenvironment that intensely favors the wound-healing process. More importantly, desired functions and features important to wound closure and healing can be achieved through elegant material selection for developing hydrogels, combining two or more different polymers, and chemical modification [2,9,14,17]. 

Thus, with water-responsive properties, hydrogels can absorb large amounts of water (from liquid or vapor phases) to form swollen 3D structures due to the presence of hydrophilic chemical groups, such as–OH, –NH_2_, –COOH, in their polymer networks and osmotic pressure [6,18,19,20]. The remarkable ability to hold an unbroken 3D structure during swelling, preventing hydrogels from dissolving in the aqueous media, is based on network crosslinking, leading to chemical stability and controlled biodegradability [6,18,21]. The swelling/deswelling behavior endows hydrogels with stimuli-responsiveness activity. Since water is the greatest component of the human body, hydrogel-based biomaterials, which can absorb and retain large amounts of water, are considered to have great potential for biomedical purposes, including wound dressings and soft tissue engineering [21]. Hydrogels are usually divided into three major classes considering the predominant original source of the material: natural polymer hydrogels (NPHs), synthetic polymer hydrogels (SPHs), and chemically modified hydrogels (or hybrid hydrogels, HHs). NPHs are made of natural polymers, often called biopolymers or semi-processed biopolymers, including cellulose and derivatives, proteoglycans, polypeptides, proteins (e.g., collagen, gelatin, fibrin, keratin), agarose, chitosan, hyaluronic acid, and others. These biopolymer-based hydrogels can be easily modified due to their abundance of chemical functionalities. Hydrogels made from natural or semi-processed biopolymers offer many advantageous characteristics over synthetic ones, such as intrinsic biocompatibility, biodegradability, non-toxicity, sustainable availability through renewable sources, abundance, and usually low costs. These features have made them the most preferred candidates for biomedical and pharmaceutical applications, including wound dressings for assisting in skin tissue healing and repair [4,7,11]. 

Among several alternatives of natural biopolymers, polysaccharides and derivatives (e.g., cellulose, chitosan, alginates, and heparin) are widely used in wound management and treatment. They often present suitable biocompatibility and resemblance to biological macromolecules and characteristics of native tissues recognized by the human body. In particular, cellulose and its derivatives have been one of the most broadly applied biopolymers for producing hydrogels, composites, and hybrids in many fields, including wound dressing, drug delivery, and tissue engineering [3,4,6]. They usually present the benefits of intrinsic biocompatibility, hydrophilicity, versatile biodegradability, abundance, low cost, and environmental friendliness. However, cellulose is insoluble in water or organic solvents, which greatly restricts its applications. Therefore, the derivatization of cellulose is achieved predominantly through their hydroxyl groups, rendering water-soluble derivatives like cellulose ethers and salts. The cellulose ether derivatives are usually characterized by water solubility, high molecular mass (Mw), and flexibility of the degree of substitution (DS), leading to broad applicability in the biomedical and pharmaceutical domains [3,4,6]. Carboxymethyl cellulose (CMC) is a cellulose ether derivative with many carboxymethyl groups grafted on the cellulose backbone by hydroxyl replacement, which has been widely used as a semi-processed biopolymer for hydrogels. The properties of CMC depend on the degree of substitution (DS) and molecular mass (Mw). CMC-based hydrogels have been developed for various applications, including soft tissue engineering, wound dressing, and drug delivery systems [3,6,22].

Nonetheless, more recently, some properties of biopolymer-based hydrogels have been improved by combining them with synthetic polymers or through chemically modified networks (hybrid hydrogels). These innovative blended composites and hybrid systems may offer attractive enhanced features in terms of functionality and properties that mimic natural tissue’s characteristics, facilitate wound healing, and repair damaged skin tissue [3,6,18,22]. Some synthetic polymers have been investigated for wound healing, including poly(vinyl alcohol) (PVA), owing to its non-toxicity, biodegradability, hydrophilicity, bio-adhesivity, and chemical resistance. PVA is a polyester with many reactive hydroxyl groups on the polymer chain, making it suitable for synthesizing hydrogel membranes for wound dressings. Additionally, PVA has high mechanical properties that support cell adhesion, propagation, and migration, but, like most synthetic polymers, PVA lacks sufficient bioactivity and the ability to accelerate the wound-healing process. Moreover, PVA is not pH-sensitive, highly needed for potential skin tissue repair applications. Therefore, PVA is not suitable to be directly applied as a bio-functional dressing for severe or chronic skin wounds, but can be combined with biopolymers, creating hybrids for improving their properties [6,7,23,24,25]. 

Hybrid hydrogels composed of carboxymethyl cellulose and poly(vinyl alcohol) have been extensively investigated for many advanced biomedical engineering applications. PVA and CMC are miscible hydrophilic polymers that are biocompatible, environmentally friendly, and biodegradable [26,27]. It is noteworthy that they have FDA (U.S. Food and Drug Administration) approval for several clinical uses, including wound dressing and pharmaceutical, nutritional, and tissue engineering applications. Thus, combining CMC with PVA associated with chemical modifications has been investigated for producing hybrid hydrogels with tailored networks, exhibiting excellent properties for developing wound dressings [26,27].

In this view, besides the versatility of combining polymers of distinct natures, the crosslinking and chemical modifications of the polymeric network offer virtually unlimited possibilities for hybrid hydrogel-based biomaterials with tunable biochemical, mechanical, and biodegradation features in promoting wound healing in skin tissue repair and restoration [28]. Essentially, hydrogels are crosslinked in one of two ways: chemically or physically. Physical gelling is reversible, as the networks are linked through either molecular entanglements or secondary forces such as electrostatic interactions, hydrophobic forces, hydrogen bonding, and ionic bonding. On the other hand, in chemical crosslinking, the polymeric networks are predominantly connected through strong, permanent covalent bonds. Physical crosslinking is considered a versatile *temporary* link, while chemical crosslinking is normally referred to as a *permanent* bond. Thus, virtually countless hydrogel networks can be tailored based on the proper selection and combination of one or more polymers with different crosslinking structures and chemical modifications [6,18].

Some bifunctional molecules have been employed as crosslinkers for covalently binding the polymer macromolecular chains in a 3D hydrophilic network to produce hybrid hydrogels. However, most crosslinkers used for modifying polymer networks are toxic, not biocompatible, and environmentally harmful (e.g., formaldehyde, glutaraldehyde, epichlorohydrin, carbodiimide, etc.), which has raised concerns regarding their applications as biomaterials. Hence, new solutions have emerged based on non-toxic crosslinkers from natural sources such as citric acid (CA) for developing environmentally and biologically friendly hydrogel biomaterials. Citric acid is non-toxic, inexpensive, and broadly used in the food industry, pharmaceutics, and biomedical applications. When heated, the carboxyl group (COOH) of CA and the hydroxyl groups (OH) of the polymer will undergo an intermolecular esterification reaction, resulting in chemical crosslinking. In this sense, modern wound dressings and skin repair substitutes could be patient designed to provide a beneficial microenvironment for promoting effective healing by avoiding contamination and controlling important properties such as hydrophilicity, surface wettability, air and electrolyte permeability, balanced wound moisture, and absorbing exudate excess [3,6,11,22].

Hence, considering all the aspects and difficulties highlighted above, it is unrealistic to expect a singular dressing strategy adopting a “one size fits all approach” to embrace all characteristics that would accomplish the requirements for the complex wound-healing process. 

Unfortunately, no such platform in the reported literature offers the prospect of permitting chronic wound-healing cascades at all phases of the dynamic biological process. The strategy to have wound dressings with tunable properties that could be tailored according to the specificities of the wound injury and patient needs is highly demanded by medical professionals [26,29]. In this view, although several reports have been published using hydrogels based on biopolymers and synthetic polymers, including CMC and PVA, there are practically unlimited possibilities for combining these polymers and modifying their networks in order to produce hybrid structures suitable for a myriad of soft tissue engineering and pharmaceutical applications [23,24,25,26,27,28,29].

**Figure 1:**
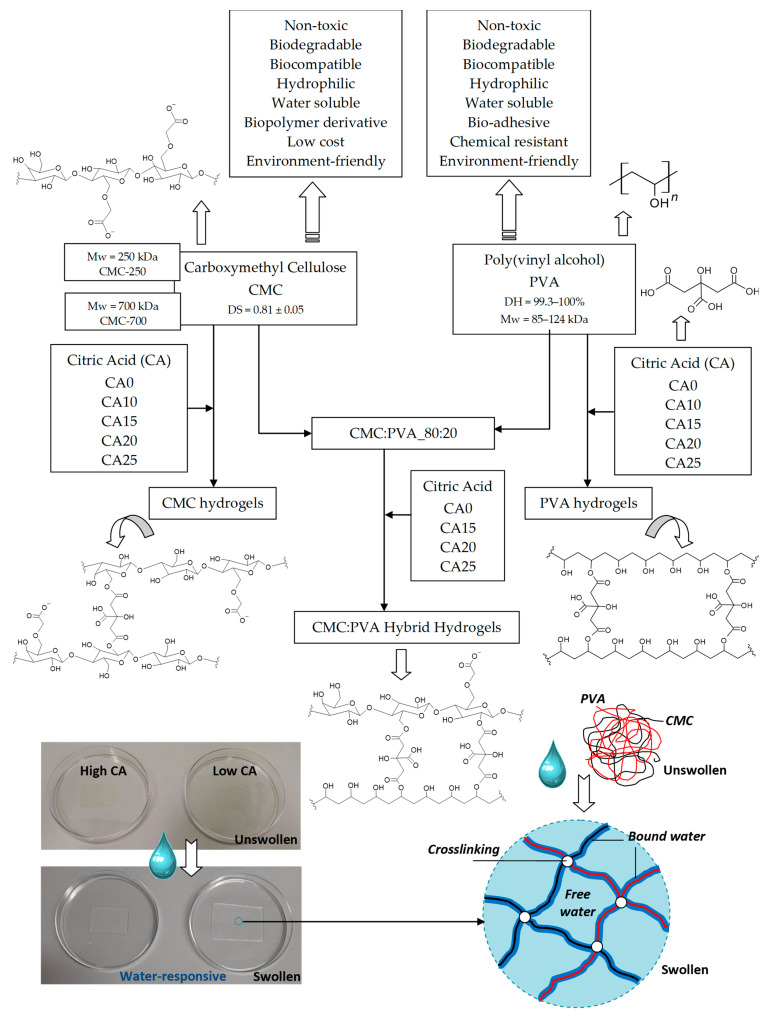
Schematic representation of the experimental design of bioengineered water-responsive CMC hydrogels, PVA hydrogels, and CMC:PVA hybrid hydrogels.

In this study, it is reported, for the first time, the design, synthesis, and comprehensive characterization of hybrid hydrogels composed of CMC, with two molecular weights (250 and 700 kDa) and degree of carboxymethylation DS = 0.81, associated with PVA, with a very high degree of hydrolysis (DH > 99%), which were physically and chemically crosslinked by different concentrations of citric acid for tuning the polymeric network. The results demonstrated that the hybrid hydrogel membranes (CMC:PVA_CA) exhibited water-responsive behavior dependent on the composition of the hybrids (i.e., polymer ratio and CA content), which were assessed using several characterization techniques, including swelling degree, gel fraction, permeability, wettability, SEM (Scanning Electron Microscopy), XPS (X-ray Photoelectron Spectroscopy), FTIR (Fourier Transform Infrared) Spectroscopy, and thermal analysis. Importantly, besides the hydrophilicity, wettability, and swelling degree characteristics, these innovative hydrogels with tailored network structures demonstrated preliminary cytocompatibility suitable for potential applications as a wound dressing and in skin tissue engineering. A schematic representation of this work’s experimental design is summarized and depicted in Figure 1.

## 2. Results and Discussion

In this study, the swelling degree (SD) parameter was used to assess the water-responsive behavior of hydrogels. In the sequence, spectroscopic studies and thermal analysis were used to evaluate the property–structure relation of the hybrid hydrogels, which were analyzed and discussed in-depth, bearing in mind their potential applications for wound dressings and skin substitutes.

### 2.1. Physicochemical Characterization of CMC Hydrogels

#### 2.1.1. Swelling Degree and Gel Fraction of CMC Hydrogels

**Figure 2:**
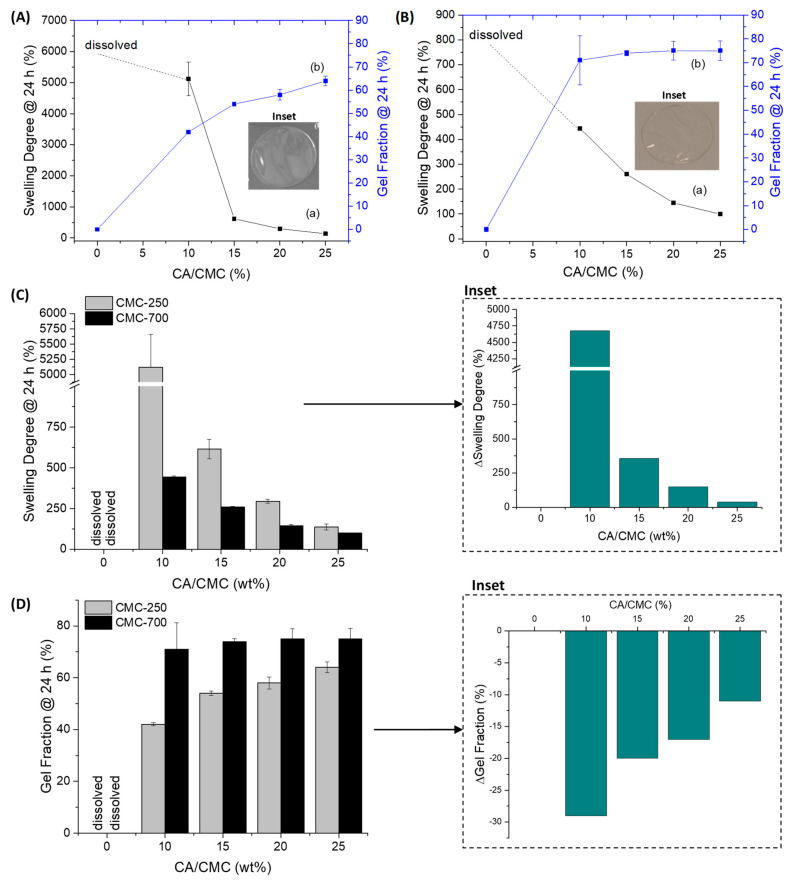
Swelling degree (**a**) and gel fraction (**b**) properties of hydrogels of CMC crosslinked with citric acid: (**A**) CMC-250 and (**B**) CMC-700. Insets: typical hydrogel membranes of CMC-250 and CMC-700 crosslinked with CA. Effect of molecular weight of CMC in (**C**) swelling degree (inset ΔSD) and (**D**) gel fraction properties of hydrogels (inset ΔGF).

Swelling degree (SD) (also referred to as swelling ratio) is widely recognized as one of the most important properties of soft biomaterials designed for wound dressing and skin tissue repair applications. The capacity of hydrogels to absorb high amounts of water while retaining their chemical stability in the swollen state is considered of pivotal relevance to maintaining wound homeostasis by balancing the moisture content and reducing the exudates in the wound site. A usual way to obtain SD is by swelling the gel in a solution liquid (in this study, deionized water, DI-water) and measuring its weight before and after immersion [6,16,30,31]. In this study, the hydrogels were membranes cast and dried, producing a relatively dense polymer network (see the digital image in Figure 1). Thus, the water uptake by the hydrogel is associated with “bound water” due to the interaction of water molecules with polymer chains and the “free water” that fills the space between the polymer network (as pores are absent) [32], leading to the dimensional changes associated with the water-responsive behavior as shown in Figure 1. Thus, Figure 2 summarizes the results of SD and gel fraction (GF) of the CMC hydrogels at a crescent concentration of chemical crosslinker (CA). 

It was observed that, for both Mw of CMC hydrogels (250 and 700 kDa), as the concentration of the chemical crosslinker (CA) was increased from 0 to 25%, the swelling degree decreased from fully solvated (no CA), ~5000% and 500% (10% CA, for CMC250 and CMC 700 respectively) to ~100–140% (25% CA, both CMC Mw). Without a crosslinking agent, both CMC hydrogels were water soluble after immersion for 24 h in DI-water. At this condition (non-chemically crosslinked), the intra- and interchain interactions (i.e., physical crosslinking) that are mostly related to hydrogen bonds were replaced by hydrogen bonds between water-CMC hydrophilic groups. In the presence of CA, the increase in water stability was expected due to the formation of chemical crosslinking through the reaction of the -OH of the CMC chain with the carboxylic groups (-COOH) of citric acid through an esterification reaction [6,33,34]. The effect of CMC Mw was more prominent at lower concentrations of the CA (10%), as there might be a relevant reduction of chain mobility for accommodation water molecules in the hydrogel network. An opposite behavior was observed for GF, which increased by raising the CA concentration. The GF increased from approximately 0% to 60% and 80% for CMC with Mw 250 and 700 kDa, respectively, at CA/CMC = 25%. However, only minor changes after the initial concentration of 10% of CA were observed for CMC-700. That provides strong evidence that the physical crosslinking at 0% of CA renders a labile network easily solvated upon immersion in water, where the chemical crosslinking stabilizes the polymeric hydrogel network, preventing it from dissolving while remaining capable of absorbing relatively high amounts of water from the medium in the swollen state of the gel network. 

It should be highlighted that although a similar trend was observed for both low and high CMC polymer Mw (250 and 700 kDa), a drastic change was verified at the lowest CA concentration, where the SD dropped 10-fold (from ~5000% to 500%) from low (250 kDa) to high Mw (700 kDa), interpreted to be associated with the lower mobility of the chain and lower degree of conformational changes upon swelling. This tendency was consistent with increasing the CA concentration but less pronounced at higher CA concentrations. 

It is interesting to note that, regarding SD, CMC-250 at CA20% reached a similar value of CMC-700 at CA15%. That is evidence that, combined with the extension of chemical crosslinking, the molecular mass parameter of the CMC biopolymer also has an important effect on the formation of the crosslinked hydrogel network, as it changes the resulting physicochemical properties. 

At a lower extension of crosslinking (CA/CMC = 10%), the hydrogels were not fully soluble, but CMC-250 polymers resulted in hydrogels with higher SD and lowered GF than CMC-700 (see ΔSwelling Degree, ΔSD = SD_CMC-250_ − SD_CMC-700_ at the same CA/CMC content) and ΔGF (ΔGel Fraction = GF_CMC-250_ − GF_CMC-700_) insets in Figure 2C,D). This behavior could be associated with physical and chemical crosslinking combined. For CMC-700, due to the higher Mw, the physical entanglement is more evident, and the constraint promoted by chemical crosslinking favored the physical stabilization of the polymeric matrix. Also, due to the lower mobility of CMC-700 chains, the formation of di-ester bonds interchains could be favored, which is endorsed by the effect of Mw in GF (CMC-250, GF ~40%, and CMC-700, GF ~70%). With the increase in the CA/CMC content, the mobility restrictions imposed by ester bond formation significantly reduce the differences between the effect of Mw in the hydrogel properties. 

Moreover, the higher degree of entanglement (i.e., which acts as physical crosslinking) for CMC-700 could be observed when comparing the thickness (*t*) of hydrogel membranes without the crosslinking agent. For CMC-700_CA0, *“t”* was equal to 37 ± 6 μm compared to 53 ± 6 μm measured for CMC-250_CA0. This could be explained by the increase in molecular weight of CMC, promoting a higher extension of polymer entanglements, while the reduction of Mw increases molecular mobility. After chemical crosslinking with CA, due to the introduction of additional covalent bonds associated with the esterification reactions, a reduction in physical dimensions was observed for both hydrogels (23 ± 6 μm and 43 ± 6 μm CMC-700_CA25 and CMC-250_CA25, respectively) [6]. This effect was assigned to the shrinking of the hydrogel network by chemical crosslinking of CMC chains. As shown by the typical digital images in the Figure 2A,B inset, the hydrogels, regardless of Mw and CA content, were uniform, optically transparent, and flexible. Thus, these findings demonstrated the important effect of polymer molecular weight and chemical crosslinking on the physicochemical stability of the hydrogels in aqueous media. The chemical stability of the hydrogel is required for wound dressing applications to assist in the healing process by filling the irregular shape of the wound injury while offering a balanced moisture absorption environment.

#### 2.1.2. FTIR Spectroscopy Analysis of CMC Hydrogels

**Figure 3:**
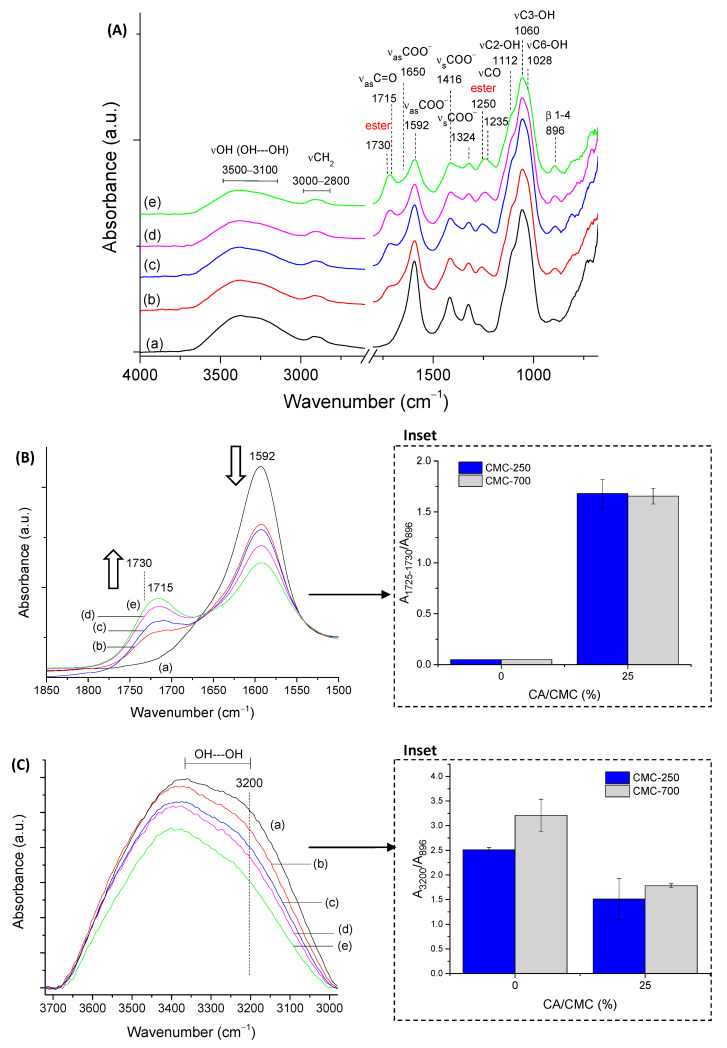
(**A**) FTIR spectra of CMC-250 crosslinked with different amounts of citric acid: (**a**) CA/CMC = 0%, (**b**) CA/CMC = 10%, (**c**) CA/CMC = 15%, (**d**) CA/CMC = 20%, and (**e**) CA/CMC = 25%. Evolution of CMC bands with increasing citric acid content: (**B**) 1850–1500 cm^−1^ range associated with ester bonds formation (inset: A_1730_/A_896_ ratio; arrows indicate the trend) and (**C**) 3700–3000 cm^−1^ range associated with hydrogen bonds’ consumption (inset: A_3200_/A_896_ OH interchain band).

FTIR spectroscopy was used to assess the CMC biopolymer chemical groups and investigate the formation of hydrogel crosslinked networks with CA. CMC-250 was selected for FTIR analysis of the main groups and of the effect of crosslinking to avoid redundancy. Then, the major effects of the higher polymer molecular weight were presented related to crosslinking compared to CMC-250. Thus, in CMC-250 spectra (Figure 3A(a)), the region could be observed in the range of 3500–3100 cm^−1,^ which is attributed to the stretching vibrations of the O-H bonds. The C–H stretching region ranges from 3000–2800 cm^−1^. The region between 1800–1200 cm^−1^ overlaps contributions from carboxylates (–COO^−^ bands at 1650 cm^−1^, 1592 cm^−1^, 1416 cm^−1,^ and 1324 cm^−1^), C-H (1370 and 1267 cm^−1^), and OH (1233 cm^−1^) bending vibrations, and symmetric (1203 cm^−1^) and asymmetric (1152 cm^−1^) stretching modes of the pyranose ring (C-O-C). The C–O vibrations from primary (C6-OH at 1028 cm^−1^) and secondary alcohols (C2-OH at 1112 cm^−1^ and C3-OH at 1060 cm^−1^) were also detected, and the vibration of glycoside bonds (β1-4) was observed at 895–900 cm^−1^. These findings showed the most important chemical groups of the CMC biopolymer, which has the typical saccharide rings linked by ether bonds, hydroxyls, and grafted carboxymethyl groups by partial substitution of OH groups [35]. 

Regarding the chemical crosslinker, citric acid possesses three carboxylic groups (−COOH, two terminal, and one central) and one C−OH group, all active as hydrogen bond-donating functional groups. The pKa’s for CA and deprotonated CA is 3.13, 4.76, and 6.40 [36]. The most characteristic of CA bands in FTIR (not shown) are (a) stretching vibrations of OH bonds and CH bonds in the 3500–3000 cm^−1^ range; (b) the C=O stretching from 1720 cm^−1^ to 1695 cm^−1^, depending on the degree of protonation and hydrogen bonding; (c) the νC-OH at 1180–1100 cm^−1^; and (d) the vibration at about 780 cm^−1^ which is assigned to CH_2_ rocking [37].

The comparison of pure CMC with crosslinked hydrogels (Figure 3A(b–e)) demonstrated the pH-sensitive behavior of this polymer. Carboxylates (–COO^-^ bands at 1650 cm^−1^, 1592 cm^−1^, 1416 cm^−1^, and 1324 cm^−1^) and carboxylic acid (–COOH bands at 1715 cm^−1^ and 1235 cm^−1^) were observed mostly due to the protonation–deprotonation of the carboxylic groups (R-COOH/R-COO^-^) in the CMC polymer chains during the acidification promoted by citric acid. The reduction of the band of carboxylates at 1592 cm^−1^ and the increase of the band at about 1715 cm^−1^ of carboxylic acid demonstrated this feature. The ester bands (R1-COO-R2) formed in the chemical crosslinking reaction between OH groups from CMC and carboxylic groups from CA, schematically depicted in Figure 1 (CMC hydrogels), are also observed in this spectra range. An analysis of peaks in the region between 1730–1700 cm^−1^ of as-synthesized samples (pH ~ 4.0 ± 0.2) and after equilibrium for 24 h in DI-water (pH ~5.5 ± 0.2, above pKa of CMC [38]) indicated that carboxylic bands from protonated CMC and citric acid are overlapped at about 1715–1700 cm^−1,^ and ester bonds at 1730–1725 cm^−1^ (Figure S1A, Supplementary Material). Moreover, the relative increase of these bands related to the CA content is depicted in Figure 3B (the arrows in the spectra indicate the trend of band increase/decrease). 

The crosslinking reaction was detected by the increase in the intensity in the region associated with ester bonds at 1250 cm^−1^ (νC-O), which is nearer to the band related to analogous vibration of COOH in CMC (~1230–1240 cm^−1^) (Figure S1B—Supplementary Material). 

Regarding the stretching vibrations of the O-H bonds (Figure 3C), it could be observed that crosslinked CMC hydrogels showed a relative decrease in intensity of -OH peak at approximately 3400–3200 cm^−1^, which was associated with the partial disruption of intra- and interchain hydrogen bonds of CMC [39] and also the consumption of OH during the crosslinking due to the chemical esterification reaction with citric acid [6,33,40].

A more detailed analysis of these regions in terms of the physical and chemical crosslinking, comparing CMC-250 to CMC-700, is depicted in the insets of Figure 3B,C based on the ratio of the absorbance of the band under analysis and the reference band of β1-4 glycoside bond at 896 cm^−1^ (A_896_). In the absence of CA, a higher degree of physical entanglement could be noted for CMC-700 using the bands related to inter- (3360–3200 cm^−1^) and intrachain (3330 cm^−1^ and 3270 cm^−1^) hydrogen bonds (>A_band_/A_Ref_) as a consequence of the higher Mw, as previously discussed. Upon CA crosslinking, the reduction of hydrogen bonds and depletion of hydroxyls reduce the absorbance of these bands and increases the absorbance of ester bands at 1730 cm^−1^. It is noteworthy that a similar degree of crosslinking was indicated at CA 25%, as projected by SD measurements. In addition, the smaller reduction in the intensity of the hydrogen bonds for CMC-250, even at the highest degree of crosslinking (CA/CMC = 25%), could be ascribed to the higher mobility associated with smaller Mw that favors the development of OH---OH interactions involving the remaining functional groups of CMC and CA. Supporting the results of the previous section, these findings endorsed the chemical modifications of the CMC hydrogel networks by different extensions of chemical crosslinking, which has tuned the physicochemical properties, including SD and GF. 

#### 2.1.3. Thermal Analysis and XPS Spectroscopy of CMC Hydrogels

**Figure 4:**
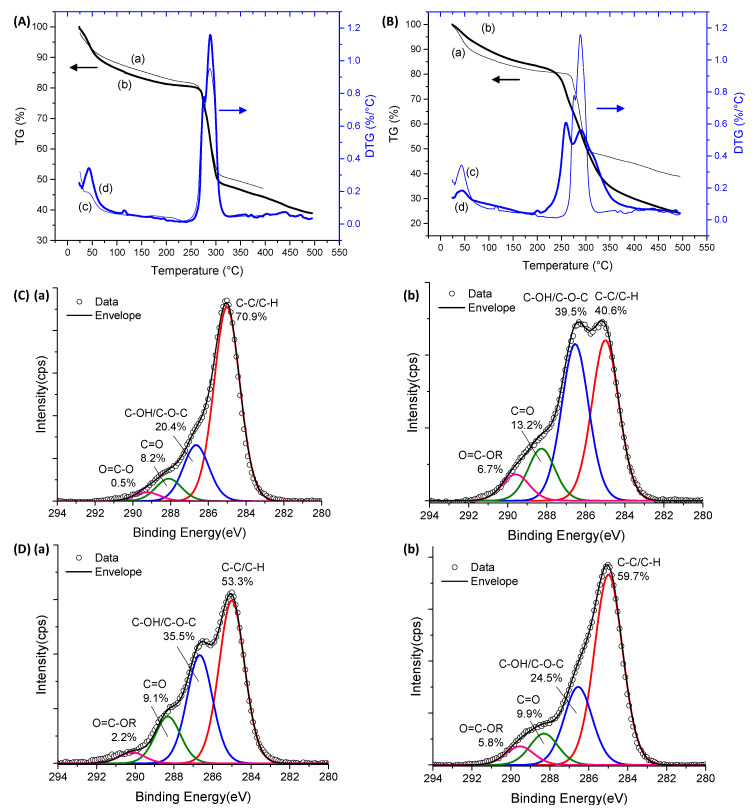
TG (**a**,**b**) and DTG (**c**,**d**) analysis of (**A**) CMC-250_CA0 (—) and CMC-700_CA0 (**—**) and (**B**) CMC-700_CA0 (—) and CMC-700_CA25 (—) hydrogels. XPS analysis of C 1s region for (**C**) CMC-250_CA0 (**a**) and CMC-250_CA25 (**b**) and (**D**) CMC-700_CA0 (**a**) and CMC-700_CA25 (**b**) hydrogels.

Thermal analysis and XPS surface chemical characterization techniques were used for further accessing the modification of the CMC polymeric network by the extension of chemical crosslinking. Thermogravimetric (TG, curves (a) and (b)) and derivative thermogravimetric (DTG, curves (c) and (d)) profiles for CMC-250 and CMC-700 with and without a crosslinking agent are exhibited in Figure 4A,B and Figure S2—Supplementary Material.

For pure CMC (Figure 4A) and crosslinked hydrogel (Figure 4B and Figure S2, Supplementary Material) structures, two main stages of mass loss were observed up to 500 °C. The first is related to water loss and can be separated into two events. Primarily, the removal of moisture up to 110 °C was observed. In the sequence, the vaporization of water molecules involved in hydrogen bonds with hydrophilic OH and COONa/COOH groups of polymer chains (110–250 °C) occurred [34,41]. Due to the high hydrophilicity of CMC polymers, a loss mass (Δm) of about 20% was observed below 250 °C. For the crosslinked hydrogels in comparison to non-crosslinked hydrogels, the reduction of the mass loss associated with this water removal and narrow range of temperature associated with this event (below 200 °C) was observed. This effect was expected, as CMC crosslinking with CA occurred with the consumption of hydroxyl groups, resulting in a smaller number of hydrophilic groups available to interact with the water molecules. The second region of mass loss was observed between 250 and 320 °C for pure CMCs and 210–400 °C for crosslinked hydrogels assigned to the thermal events of exothermic degradation of polymers. For CMC (CMC-250_CA0 and CMC-700_CA0), this region in DTG curves presented a small peak at 270–275 °C and the main decomposition peak at approximately 285–290 °C. CMC-700_CA25 presented lower thermal stability than non-crosslinked analogs, involving a first event at about 260 °C and a second event at a similar temperature of pure CMC (T_max_ = 290 °C). Similar behavior was observed for crosslinked CMC-250 (Figure S2—Supplementary Material). In addition, at 500 °C, the total weight loss was about 60% and 75% for CMC-700 before and after crosslinking, respectively.

These differences could be assigned to the distinct interactions between polymeric chains in crosslinked and non-crosslinked samples, which involve reducing the number of hydrogen bonds’ intra- and interchains and introducing ester bridges. Regarding the thermal profile, there was no significant difference for CMC as a function of Mw 250 and 700 kDa. Hence, the thermal analysis corroborated the FTIR results, demonstrating that the extension of chemical crosslinking by CA modified the CMC hydrogel networks. Consequently, major changes were detected in the thermal profiles and events.

XPS spectra of the C 1s region before crosslinking reactions (CA_0) presented the characteristic peaks of CMC (Figure 4C(a),D(a) for CMC-250 and CMC-700, respectively). Peaks at 285.0, 286.6, 288.1, and 289.2 eV can be assigned to C–H and C–C bonds (hydrocarbons), C–OH (alcohol), and C–O–C (ether) bonds, C=O (carbonyl) bonds, and O=C–O bonds (acid and esters) [35,42,43]. 

For CMC-700, the peak of O=C–O is shifted to higher binding energy (left-hand side), which is speculated to be due to a higher density of hydrogen bonding as observed in FTIR analysis [44].

Upon CA addition and crosslinking reaction, for both Mw (Figure 4C(b),D(b)), the O=C–O peak area increased considerably compared with CMC hydrogel without citric acid, confirming the occurrence of ester bond formation and the presence of CA in the system. In addition, CMC-700_CA25 hydrogel depicted a clear reduction in the peak area associated with the C–OH/C–O–C bond and a relative increase of the band associated with C–H/C–C binding energy. On the contrary, for CMC-250_CA25, the contribution of the C–OH/C–O–C peak was increased at the surface of the hydrogel. This different trend may be attributed to a new conformation of the CMC-250 upon adding CA and thermal treatment to reduce the *free energy* favored by this system’s higher mobility/lower viscosity. 

Based on the results of this section devoted to CMC hydrogels, the chemical crosslinking through the formation of ester bonds was demonstrated, reducing the swelling degree with the increase in CA content, which was associated with the consumption of the hydroxyl hydrophilic groups available for interacting with the water molecules. Moreover, the significant effect of the Mw of CMC in the water-responsive behavior of hydrogels was verified, which was mostly ascribed to the mobility of the chains affecting the development of physical crosslinking between chains. Analogously, the formation of ester bonds resulted in the reduction of the thermal stability of the polymer network.

### 2.2. PVA Hydrogel Characterization

#### 2.2.1. Swelling Degree and Gel Fraction of PVA Hydrogels

**Figure 5:**
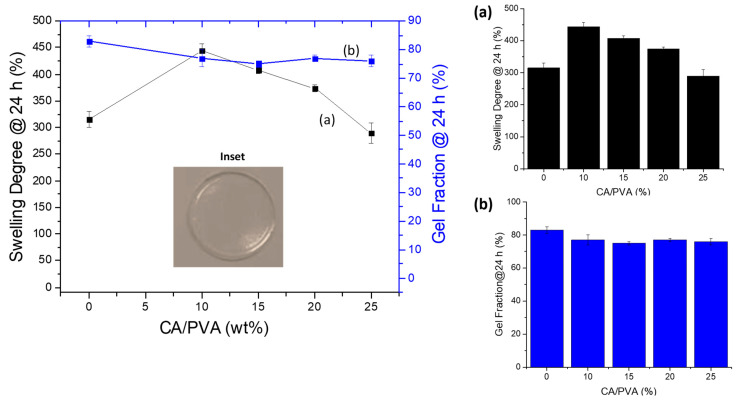
Swelling degree (**a**) and gel fraction (**b**) properties of hydrogels of PVA crosslinked with citric acid (inset: digital image of PVA_CA25).

The water uptake and stability properties of PVA-based hydrogel are presented in Figure 5. Without adding the crosslinking agent, PVA hydrogel was not fully solvated upon immersion in water and had a swelling degree value of about 315%. After adding a small CA content (CA/PVA = 10%), SD WA increased to 450%. This effect was assigned to the interactions between PVA chains, mostly hydrogen bonds, which were replaced by ester bonds between the citric acid and PVA hydroxyl groups (Figure 1, PVA hydrogels). As the concentration of CA was increased, the SD slowly decreased, and only at a CA content of 25% was the water uptake smaller than the non-crosslinked PVA (SD = 290%). These results indicated that for fully hydrolyzed PVA (i.e., degree of hydrolysis > 99%), the physical crosslinking based on hydrogen bonds was more efficient in constraining swelling degree. Following a similar trend, the gel fraction parameter decreased with the addition of CA and remained practically unchanged regardless of the degree of crosslinking (GF = 83%, CA0; and GF = ~77%, CA10-25). 

Based on the inset in Figure 5, it was noted that the PVA hydrogel was uniform and transparent. The membranes were flexible, and the average thickness of the membranes was reduced by approximately 30% (*t* = 33 ± 6 μm for PVA_CA0 and *t* = 23 ± 6 μm for PVA_CA25) with crosslinking at CA/PVA = 25%.

#### 2.2.2. FTIR Spectroscopy of PVA Hydrogels

**Figure 6:**
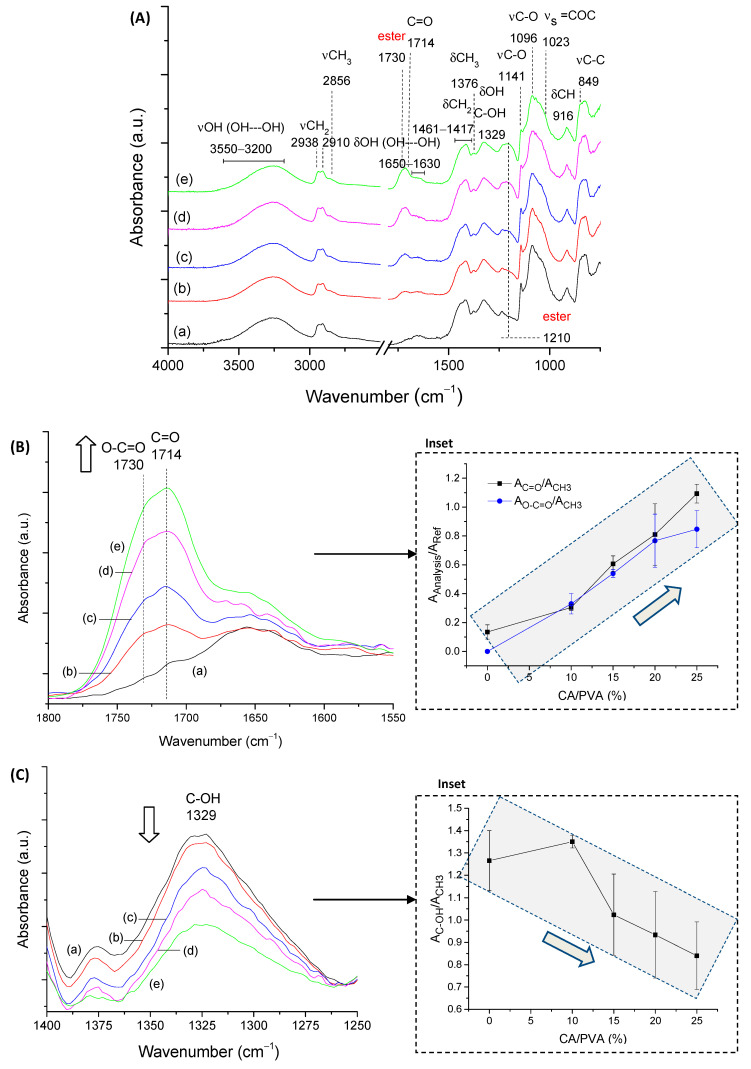
(**A**) FTIR spectra of PVA crosslinked with different amounts of CA/PVA: (**a**) 0%, (**b**) 10%, (**c**) 15%, (**d**) 20%, and (**e**) 25%. Evolution of (**B**) C=O (1714 cm^−1^) and O-C=O (1730 cm^−1^) (inset: A_C=O_/A_CH3_ and A_O-C=O_/A_CH3_ ratios), and (**C**) C-OH band (1390 cm^−1^, inset: A_C-OH_/A_CH3_ ratio). Arrows indicate the trend of the parameter/band.

The FTIR spectrum of pure PVA (PVA_CA0, Figure 6A(a)) shows the most characteristic bands of PVA and their respective assignment. The broad band observed between 3550 and 3200 cm^−1^ is associated with stretching OH from the inter- and intramolecular hydrogen bonds between PVA chains. The absorption bands observed at 2938 cm^−1^ and 2910 cm^−1^ are related to the νCH_2_ (methylene) groups. Other important bands of PVA are associated with the vibrations of hydroxyls (C–OH), which are observed at 1329 cm^−1^ (δOH) and 1141 and 1096 cm^−1^ (νC–O), and alkyl groups (δCH_2_, 1461–1417 and 916 cm^−1^). The bands at 2856 cm^−1^, 1714 cm^−1^, 1376 cm^−1^, and 1270 and 1023 cm^−1^ are associated with νCH_3_ (methyl), νC=O, δCH_3_, and ν_as_ and ν_s_ of (=C–O–C), respectively, from remaining vinyl acetate units (i.e., PVA synthesis). These bands have relatively low intensity, as the selected PVA is considered fully hydrolyzed (DH > 99%), which means that most of the acetate groups were replaced by hydroxyl groups during the saponification reaction [45]. 

Upon chemical crosslinking with CA (Figure 6A(b–e)), the appearance of the bands was observed at 1714 cm^−1^ associated with νC=O vibration from CA, and 1730 cm^−1^ (νC=O) and 1210 cm^−1^ (νC-O) assigned to the esterification reaction as presented in detail in Figure 6B. The increase in CA content in PVA hydrogels enhanced the formation of ester bridges evaluated by the evolution of the absorbance band at 1730 cm^−1^. This effect was evidenced through the ratio of the intensities associated with that vibration and the stretching vibration related to CH_3_ groups at 2586 cm^−1^ (A_O-C=O_/A_CH3_, inset in Figure 6B). This vibration related to alkyl groups remained practically unchanged, as it depends only on the DH of PVA (constant in this study). Also, the increase in the absorption of the C=O band of CA at 1714 cm^−1^ (A_C=O_/A_CH3_, inset in Figure 6B) is expected because not all carboxyl groups of CA are involved in the chemical crosslinking and, thus, are available for physical interactions through hydrogen bonds.

For PVA, the consumption of hydroxyl groups in the reaction was not followed by a noticeable reduction in the intensity of νOH from the molecular hydrogen bonds’ region centered at 3260 cm^−1^, which remained practically constant for the different CA contents (Figure S3A—Supplementary Material). This trend could be explained by the fact that each CA molecule introduces two (di-ester bridge formation) or three (monoester formation) functional groups for additional hydrogen bonds. However, the absorbance ratio for the band of C-OH of PVA at 1329 cm^−1^ reduced with the increase in the amount of citric acid to indicate the consumption of hydroxyl groups in the esterification reaction (Figure 6C).

The crystalline portion of the polymeric chains influences the intensity of the peak of PVA at 1141 cm^−1^. It is associated with forming intramolecular hydrogen bonds between two neighboring OH groups located on the same side of the plane of the carbon chain. The ratio of band intensities at 1141 cm^−1^ and 1096 cm^−1^ was evaluated and indicated a reduction in the crystallinity of PVA hydrogel with an increase in the CA content (Figure S3B—Supplementary Material). That was projected when hydrogen bonds between PVA chains were replaced by ester bonds, bridging the polymeric molecules, restraining PVA chain mobility, and weakening the driving force for crystallization [45].

#### 2.2.3. Thermal Analysis and XPS Spectroscopy of PVA Hydrogels

**Figure 7:**
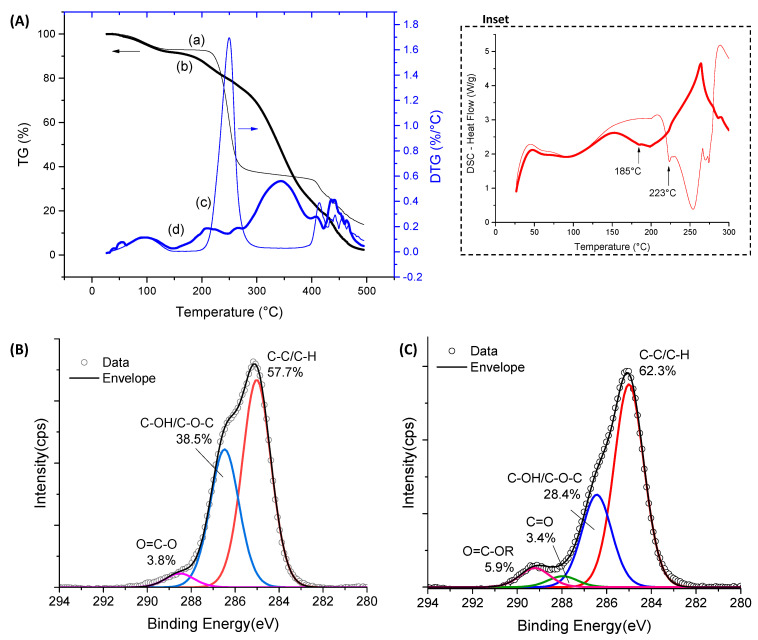
(**A**) TG (**a**,**b**) and DTG (**c**,**d**) analysis of PVA_CA0 (—) and PVA_CA25(**—**) hydrogels (inset DSC curves). XPS analysis of C 1s region of (**B**) PVA_CA0 and (**C**) PVA_CA25 hydrogels.

Thermogravimetric analysis of pure PVA (non-crosslinked by CA) displayed three major weight loss steps (Figure 7A(a)), indicated in the DTG curves. The first one, from room temperature up to 150 °C with a weight loss of about 7.1%, is associated with water loss. As previously described for CMC (previous section), as expected, the presence of water molecules adsorbed and strongly bound to the hydrophilic hydroxyl groups. The second step was observed after the melting of the polymer at 223 °C (see Differential scanning calorimetry, DSC, curve (a) in the inset of Figure 7A), in the temperature range between 200 and 300 °C (T_max_ = 250 °C) and involved the major mass loss (Δm = 55%) of PVA decomposition. In the sequence, a further weight loss at temperatures higher than 380 °C was displayed, leaving a residue of 14% at 500 °C [46,47]. The degree of crystallinity based on the enthalpy of melting was estimated as 17% from the DSC thermogram (ΔH_melting_ = 24 J/g and ΔH_melting_ 100% crystalline = 138.6 J/g) [48]. 

After the chemical crosslinking reaction (Figure 7A(b)), the thermal analysis of PVA indicated no significant changes in the water loss up to 110 °C associated with adsorbed water but an increase in tightly bound water. This effect was related to the presence of the CA crosslinker that, even after covalent bond formation, possesses residual hydrophilic groups available for hydrogen bonding, which was indeed observed in FTIR analysis. Overlapped with this region of water removal, it could be observed that, when CA was added, the melting temperature decreased to about 185 °C, and the height of the melting peak decreased. That indicated a reduction of crystallinity in agreement with FTIR analysis [49], followed by a decomposition of about 10%. This evidenced that the crosslinking reaction of PVA with 25% of CA/PVA resulted in a decrease in the thermal stability of the polymer despite the main region of degradation occurring in the range of 250–420 °C (Δm ~ 61%, T_max_ = 345 °C), which is shifted to higher temperatures in comparison to pure PVA. With a further temperature increase, at 500 °C, the sample was more than 96% degraded. 

Figure 7B shows the C ls spectrum of non-crosslinked PVA, which presented two major peaks at 285.0 eV and 286.5 eV attributable to the C–H/C–C and C–OH species of PVA unit [43,50], respectively. Also, a small peak centered at 288.5 eV is assigned to the ester (O–C=O) species of vinyl acetate units in PVA [51]. For crosslinked samples (CA_25, Figure 7C), a clear reduction of the peak area associated with the C–OH bond was observed, which is consistent with the occurrence of crosslinking reactions involving hydroxyl groups of CMC, correlated with an increase in the peak related to ester groups (O–C–OR) and citric acid (C=O and O–C=O) bonds. Hence, the thermal analysis associated with the XPS and FTIR results validated that the extension of chemical crosslinking modified the PVA hydrogel networks by forming covalent bonds, rendering stable and flexible polymer networks with water-responsible characteristics which are relevant for prospective wound dressing applications. 

### 2.3. Characterization of CMC:PVA Hybrid Hydrogels

#### 2.3.1. Swelling Degree and Gel Fraction of CMC:PVA Hybrid Hydrogels

**Figure 8:**
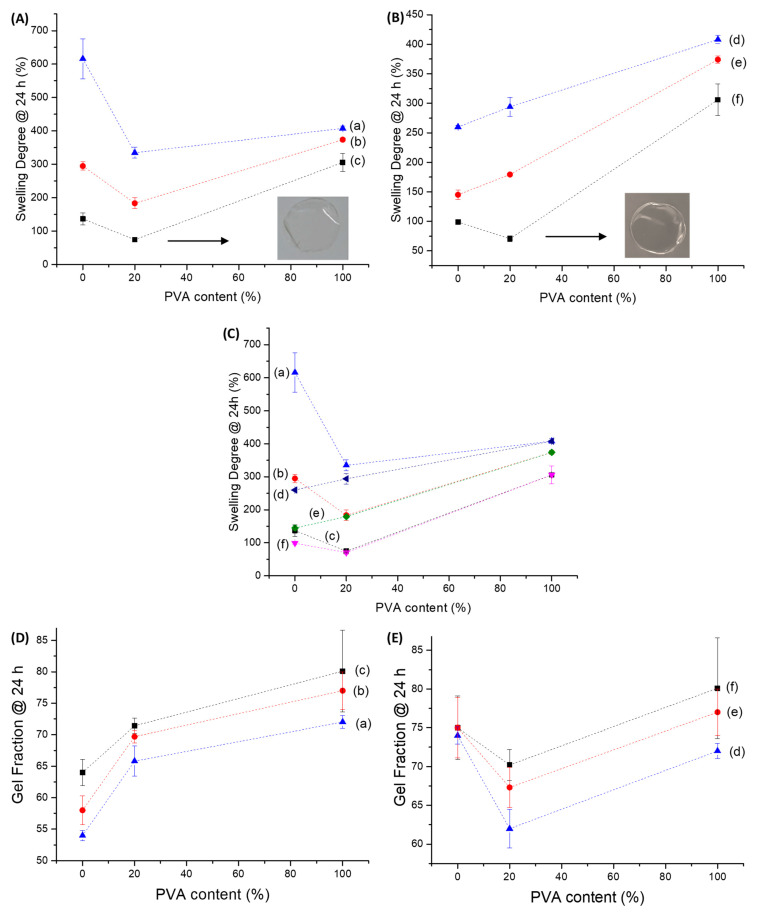
Swelling degree ((**A**) CMC-250:PVA, (**B**) CMC-700:PVA, and (**C**) effect of CMC Mw. Inset: digital images of hybrids at CA25) and gel fraction ((**D**) CMC-250:PVA and (**E**) CMC-700:PVA) properties of hybrids crosslinked with different amounts of CA: (**a**) CMC-250:PVA_CA15, (**b**) CMC-250:PVA_CA20, (**c**) CMC-250:PVA_CA25, (**d**) CMC-700:PVA_CA15, (**e**) CMC-700:PVA_CA20, and (**f**) CMC-700:PVA_CA25.

After performing the full characterization and analysis of CMC and PVA components separately for forming hydrogels, they were amalgamated to produce innovative hybrid networks with suitable properties focusing on wound dressing applications. Thus, the results of swelling degree and gel fraction for CMC:PVA hydrogel hybrids are depicted in Figure 8. A reduction of SD was observed as the concentration of CA crosslinker was increased, following a similar trend previously discussed for pure hydrogels of unblended polymers CMC and PVA. Moreover, in the absence of CA, all hybrid hydrogels were fully solvated, independent of the crosslinking content and Mw of CMC. 

Regarding the effect of the Mw of CMC, for CMC-250 (Figure 8A), a reduction of SD of hybrids was detected compared to pure polymer, independent of the CA/polymer content. This effect was assigned to a higher miscibility/interaction between PVA and CMC polymers with contributions of both physical and chemical interactions. 

On the contrary, for CMC-700 (Figure 8B), at CA/polymer of 15% and 20%, the SD values mostly followed the “law of mixtures”, with blends presenting SD values consistent with the sum of contributions of each polymer (straight line). 

However, this effect was overcome at the highest crosslinker concentration (CA/polymer = 25%), indicating increased interactions/crosslinking between polymers. It may be explained by the higher viscosity/lower mobility/higher entanglement of CMC chains due to the high molecular weight (700 kDa) and the eventual plasticizing effect of citric acid at higher concentrations [52,53].

When comparing the SD values of blends at the same CA/polymer content (Figure 8C), it can be observed that they were equal (CA/polymer = 25 and 20%) or tended to be similar (CA/polymer = 15%) independent of the Mw of polymer (250 or 700 kDa). This behavior indicated that at high CA content, the extension of crosslinking of hydrogel networks evaluated by the SD parameter was not primarily affected by the Mw of CMC. This trend can be explained by the fact that the average number of functional groups available for the crosslinking chemical reaction was the same, as the degree of substitution of both CMCs was similar (DS~0.8). 

GF is considered an assessment of the chemical stability of the hydrogel network in the water medium, resulting in the formation of the swollen matrix while not dissolving. Thus, as expected, as described in previous sections for pure hydrogels, the results of the GF of CMC:PVA hybrids demonstrated an opposite behavior to that observed for SD. The effect of increasing the concentration of CA/polymer in CMC:PVA hybrids produced more stable hydrogels (i.e., a higher GF) ascribed to a higher extension of chemical crosslinking (Figure 8D,E). 

The addition of PVA increased the GF of the hybrids compared to pure CMC-250 hydrogels, with an opposite behavior observed for CMC-700. These results indicated that the interactions and chemical crosslinking between CMC-250 and PVA may have favored the overall stability of the hydrogel network. Conversely, adding PVA to CMC-700-based hybrids resulted in a lower GF for polymer mixtures compared to each polymer, i.e., PVA and CMC-700 hydrogels. This result was interpreted as the effect of disruption of the CMC-700 polymer chain entanglement of high molecular weight by the insertion of PVA forming the hybrid network. This effect of reduction of the GF was observed after the chemical crosslinking with CA forming ester bonds, even at the highest concentration of CA. So, different from CMC-250, the insertion of PVA into the CMC-700 network was not favorable to the chemical stability of the hybrid hydrogel produced. 

The digital images presented in the insets of Figure 8A,B related to CMC-250:PVA_CA and CMC-700:PVA_CA, respectively, showed that the hybrid hydrogels were transparent and uniform, without evidence of phase separation or segregation. Analogously to pure CMC and PVA hydrogels of previous sections, hybrid hydrogels (CMC:PVA_CA) demonstrated physicochemical stability in water media dependent on the extension of network crosslinking by citric acid. Thus, they offered intermediate features and properties due to the contributions of both polymers, which are considered preliminarily compatible for potential application as wound dressings, where the water absorption can be tailored for the specificities of the wound injury. 

#### 2.3.2. FTIR Spectroscopy of CMC:PVA Hybrid Hydrogels 

**Figure 9:**
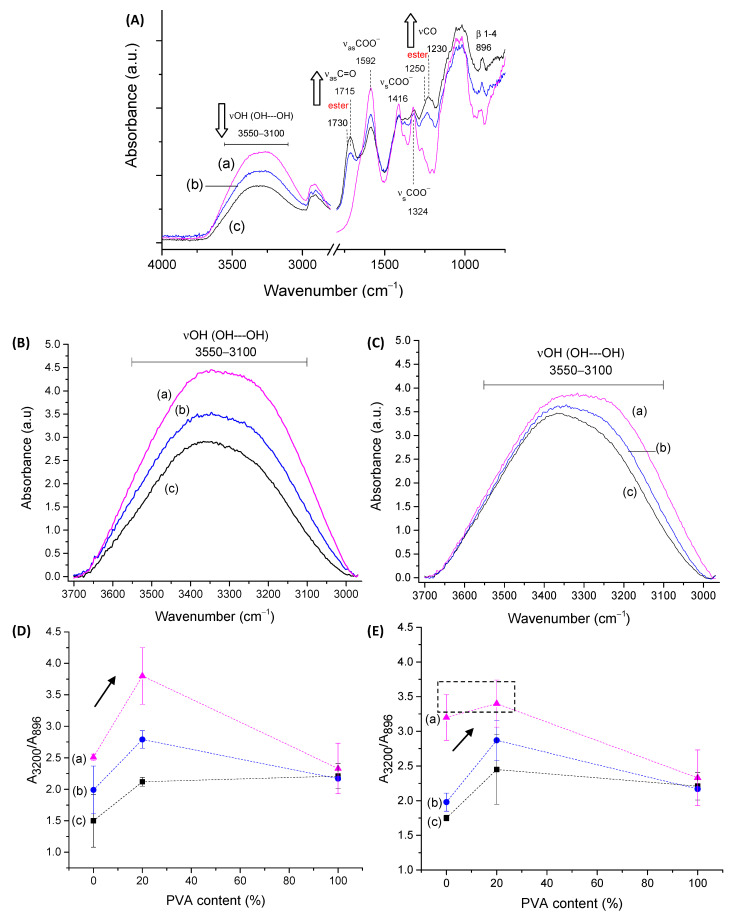
(**A**) FTIR spectra of CMC-250:PVA with (**a**) CA0, (**b**) CA15, and (**c**) CA25. Detail of FTIR spectra in the range of 3700–3000 cm^−1^ for (**B**) CMC-250:PVA and (**C**) CMC-700:PVA ((**a**) CA0, (**b**) CA15, and (**c**) CA25). Evolution of the ratio of bands A_3200_/A_896_ as a function of PVA content for (**D**) CMC-250:PVA and (**E**) CMC-700:PVA ((**a**) CA0, (**b**) CA15, and (**c**) CA25).

First, based on the FTIR spectra of the hybrids for both CMC samples with PVA presented in Figure 9A, it can be observed that they are mostly dominated by the bands of cellulose-derivative (as previously presented in Figure 3A), which is the main component of the system with 80% polymer content (PVA = 20%). Upon the chemical crosslinking reaction, the same trends observed for CMC pure hydrogels were observed, as follows: (i) reduction of absorbance in the region of 3600–3000 cm^−1^ associated with hydrogen-bonded molecules; and (ii) increase in the regions at 1750–1700 cm^−1^ and 1250–1230 cm^−1^ that by overlapping the vibrations related to ester bond formation and CA and CMC remained carboxyl/carboxylate groups (arrows in spectra indicate band increase/decrease trend). To avoid redundancy, only the main peaks were presented in Figure 9A. 

When comparing the effect of the Mw of CMC in the hybrid formation, the analysis of the region of the spectra associated with hydrogen bonds (Figure 9B–E) reveals some important features of hybrid formation. It was noted that for CMC-250:PVA hybrid, higher miscibility/interactions between polymers were observed due to a significant increase in the ratio A_3200_/A_896_ in the absence of crosslinking agent (CA0) assigned to an enhancement of the hydrogen bonds in the system. For CMC-700:PVA, no significant change in the ratio A_3200_/A_896_ was detected compared to the pure CMC hydrogel analog. Upon CA addition, a significant change in this ratio for the CMC-250-based hybrid was also observed, indicating the contribution of ester bonds to forming its hybrid structure. On the contrary, CMC-700:PVA showed a relatively smaller reduction in this OH intra-/interchain band vibration. However, the values of A_3200_/A_896_ tended to be similar (2.1 ± 0.1 and 2.5 ± 0.5 for CMC-250:PVA_CA25 and CMC-700:PVA_CA25, respectively). It was noteworthy that for CMC-700 hybrids with citric acid, for both CA15 and CA25 amounts, the increase in this absorbance band was detected when compared to pure polymer hydrogels. This effect was associated with increased interactions between CMC and PVA, mediated by the plasticizing effect of CA and its ability to promote hydrogen bond formation.

These results agreed with the previous analysis of SD of hydrogel hybrids that indicated higher miscibility for lower molecular weight CMC and increased interactions/miscibility for higher Mw CMC upon CA addition. Compared to SD results (Figure 8A,B), these results indicated that physical interactions directly interfere with the formation of the hydrogels. However, the chemical stability in the water medium was predominantly achieved by forming ester bonds, even when PVA was added to the system.

#### 2.3.3. Thermal Analysis and XPS Spectroscopy of CMC:PVA Hybrid Hydrogels

**Figure 10:**
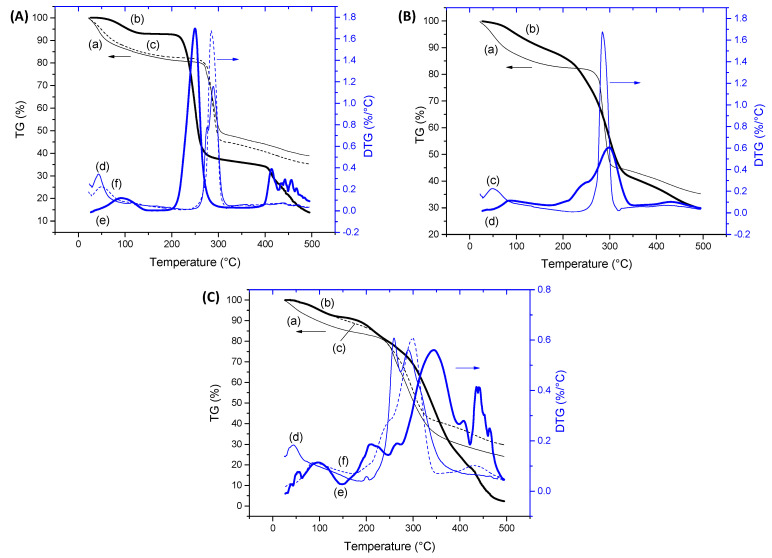
(**A**) TG (**a**–**c**) and DTG (**d**,**f**) analysis of CMC-700_CA0 (—, solid line), PVA_CA0 (**—**), and CMC-700:PVA_CA0 (---, dashed line) hydrogels. (**B**) TG (**a**,**b**) and DTG (**c**,**d**) analysis of CMC-700:PVA_CA0 (—) and CMC-700:PVA_CA25 (**—**) hydrogels. (**C**) TG (**a**–**c**) and DTG (**d**–**f**) analysis of CMC-700_CA25 (—), PVA_CA25 (**—**), and CMC-700:PVA_CA25 (---) hydrogels.

The TG and DTG curves of CMC-700:PVA hydrogel were presented in Figure 10A–C, compared to pure polymers with and without CA crosslinking. For the hybrids without CA (Figure 10A), the behavior was similar to the uncrosslinked pure CMC hydrogels, as it was the major constituent of the hybrid structure (80%), with only a minor increase in mass loss at 500 °C. It means that, despite the lower thermal stability of pure PVA used as a CMC-hydrogel modifier, it did not influence the values observed for non-crosslinked materials. The same thermal events were observed, i.e., the mass loss associated with water removal up to 200 °C followed by polymer degradation from 250 °C. Upon crosslinking reaction (Figure 10B), the same trend of a reduction in stability observed in the previous analysis for CMC-700_CA and PVA_CA was displayed by hybrid hydrogels, and the main stage of thermal degradation initiated at about 180 °C. 

A comparison of the thermal behavior of the crosslinked hybrid (CMC-700:PVA_CA25) with its individual components after the introduction of ester bonds (CMC-700_CA25 and PVA_CA25) is depicted in Figure 10C. It could be observed that the thermal profile at the region associated with a loss of adsorbed water (up to 100 °C) of the hybrid hydrogel followed the PVA curve. This behavior indicated the modification of the original CMC’s hydrophilic/hydrophobic behavior with the addition of PVA. In the sequence of the heating program, the degradation behavior was similar to crosslinked CMC. Thus, it is important to note that the results of chemical and thermal stability demonstrated for the CMC:PVA hybrid hydrogels are initially suitable for applications in wound dressing and skin tissue engineering, where no relevant changes in properties should occur, and no thermal degradation is expected under the clinical conditions.

**Figure 11:**
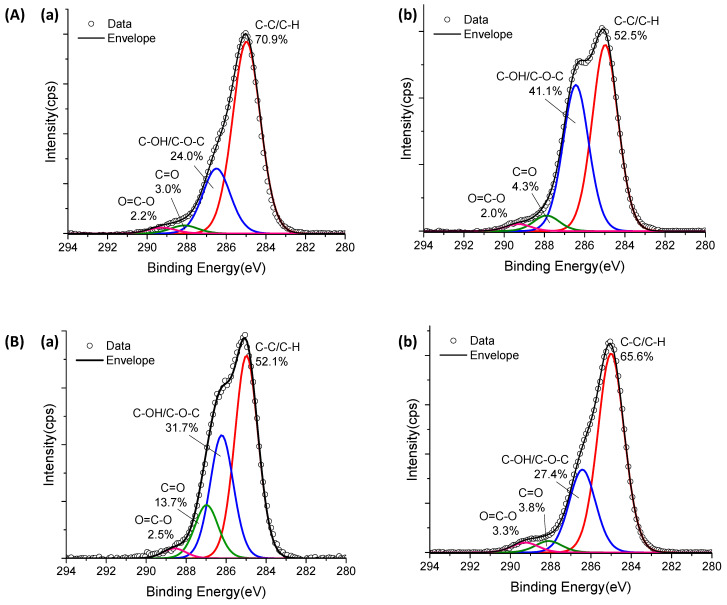
XPS analysis of C 1s region of (**A**) CMC-250:PVA and (**B**) CMC-700:PVA hybrids with CA/polymer content (**a**) 0% and (**b**) 25%.

Regarding the XPS analysis (Figure 11), the C–OH/C–O–C and C–C/C–H bonds behaved similarly to pure CMC upon crosslinking, which was associated with the higher mobility/lower viscosity of CMC-250 to achieve lower free energy. However, the presence of CA and the formation of ester bonds at the surface were not clearly observed for both CMCs, probably due to the new conformation associated with the PVA network modifier.

#### 2.3.4. Morphological Analysis of CMC:PVA Hybrid Hydrogels

**Figure 12:**
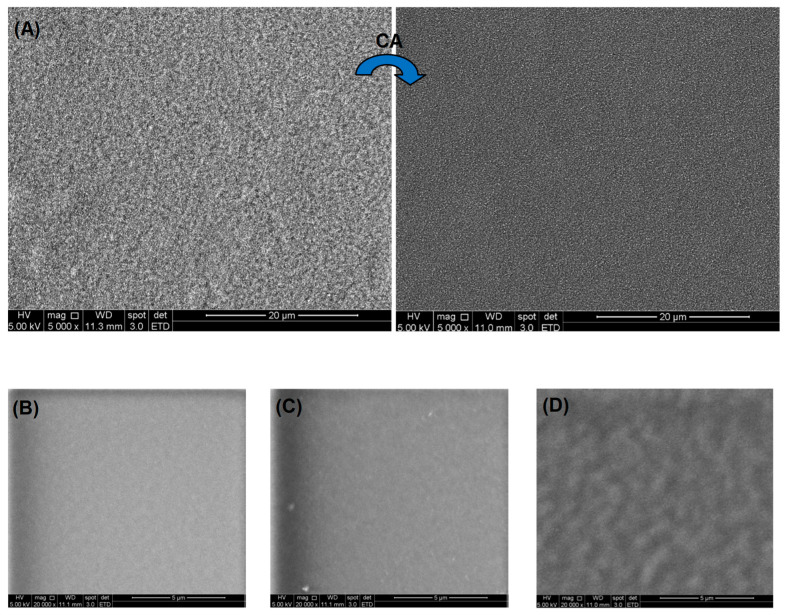
(**A**) SEM images of CMC-700:PVA blend before (**left side**, CA0) and after (**right side**, CA25) crosslinking reactions (5000×, scale bar = 20 μm). SEM images of crosslinked (**B**) CMC-250_CA20, (**C**) PVA_CA20, and (**D**) CMC-700:PVA_CA25 (20,000×, scale bar = 5 μm).

Regarding the morphological features, SEM images of the non-crosslinked (CMC-700:PVA_CA0, Figure 12A left side) hydrogels exhibited a coarse morphology with granular features that became significantly refined upon citric acid crosslinking (CMC-700:PVA_CA25, Figure 12A right side) as a result of a more uniform organization of the polymeric chains. When comparing images of crosslinked samples of individual polymers (Figure 12B, CMC-250, and Figure 12C, PVA) with CMC-700:PVA_CA25 hybrid (Figure 12D) at higher magnification (20,000×), it can be observed that the pure materials are relatively more uniform than the mixture of polymers at the microscopic level. Nonetheless, no segregation or failure of miscibility was observed with homogenous surface morphology. The uniformity and absence of segregations or voids of these hybrid hydrogels are considered highly promising when directed toward wound dressing applications, where a more consistent behavior of the clinical product is expected.

#### 2.3.5. Wettability, Hydrophilicity, and Permeability of CMC:PVA Hybrid Hydrogels

**Figure 13:**
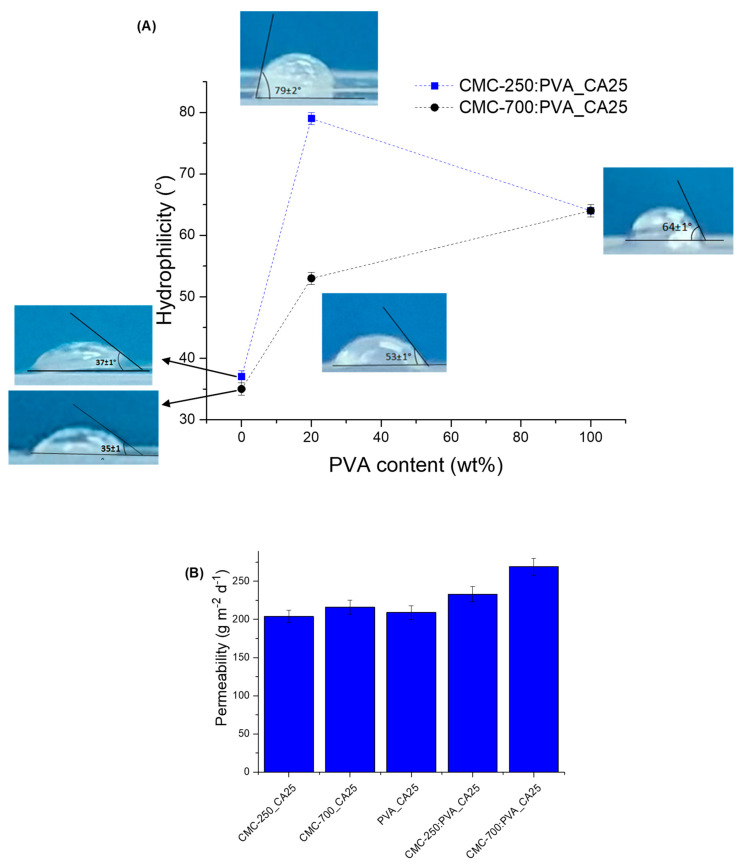
(**A**) Evaluation of wettability of crosslinked hydrogels (CA/polymer = 25%) based on the static contact angle measurements (sessile drop method), including typical digital images obtained for different systems under evaluation. (**B**) Permeability of crosslinked hydrogels.

The hydrophilic/hydrophobic behavior of crosslinked pure polymer hydrogels and hybrids (CA/polymer = 25 wt%) was evaluated based on static contact angle (sessile drop, Figure 13A) using hydrogels in a “dry state” (“as-synthesized ”). The results indicated higher hydrophilicity for pure hydrogels with contact angles of 37° and 35° for CMC-250_CA25 and CMC-700_CA25, respectively. These values were very similar regardless of the differences in Mw and SD. This can be ascribed to the similar content of the hydrophilic and reactive groups in both polymers (DS ~ 0.81) available for chemical crosslinking, hydrogen bonds, and other weak interactions. Lower hydrophilicity was measured for PVA polymer, compared to CMC (contact angle = 64°). Interestingly, the PVA hydrogel at CA/PVA = 25 wt% presented higher SD (306%) compared to CMC hydrogels (137% and 99% for CMC-250_CA25 and CMC-700_CA25, respectively). The lower hydrophilicity (i.e., higher contact angle) of PVA hydrogels can be related to the sum of ester bond formation (crosslinking reaction) and hydrogen bonds between hydroxyl groups of PVA, resulting in physical crosslinking intra- and inter-chains. Also, the occurrence of hydrogen bonds between OH groups from PVA with carboxylates and alcohol groups from CA cannot be neglected, as indicated by FTIR results (Figure S3A—Supplementary Material). These interactions may have reduced the number of hydrophilic groups available at the surface to decrease water tension (γ_water_). A similar trend was observed for the CMC-250:PVA_CA25 hybrid with a contact angle of 79°, higher than that measured for CMC-700:PVA_CA25 hydrogel (53°). These contact angle values measured for the hybrids agree with the optimum values reported for promoting cell attachment and proliferation (55–85°) [54].

The difference in adding PVA to CMC on the contact angle can be interpreted as the overall balance of interactions of the chemical groups and polymer backbone chain conformations towards finding the lowest free energy state. Therefore, due to the higher mobility/lower viscosity associated with the CMC with lower Mw (250 kDa), upon blending with PVA and chemical crosslinking, a great number of hydrogen bonds and other weak interactions are expected to be formed in the hybrid hydrogel network (“bulk”). Thus, in an ordered and cooperative way governed predominantly by hydrogen bonding in the hydrogel network, that would have reduced the hydroxyls available, increasing the surface energy and contact angle, which has been reported for PVA films when the hydrolysis degree is >98% [55]. A similar effect was not evidenced for CMC-700-PVA hybrids, as the higher CMC Mw may have partially limited all the conformational changes needed for forming the hydrogen bonds and chemical crosslinking. Moreover, these results agree with the thermal analysis findings that indicated a reduction of hydrophilicity of the CMC in the presence of PVA based on a reduction of the mass loss associated with adsorbed water (Figure 10C). Here, it suggested that the thermodynamic balance combined with kinetics governed the formation of internal hydrogen bonds compared to the surface. 

The permeability results obtained for hybrids are presented in Figure 13B. They indicate that the hydrogels presented water vapor transmission (WVT) from 200 to 260 gm^−2^d^−1^. 

These results are within the range of WVT values for normal (healthy) skin tissue (~200 gm^−2^d^−1^) and commercial wound dressings (76–9360 gm^−2^d^−1^) [56]. This characteristic may be related to the morphology of the samples observed by SEM (Figure 12D). The WVT was lower for a single polymer hydrogel, CMC or PVA. 

These findings regarding the surface wettability, hydrophilicity, and permeability aspects confirmed the potential suitability of the innovative hybrid hydrogels as wound dressings, where conventional dressings can maintain a balanced moist microenvironment at the wound site. That is of critical importance, considering that excessive exudates overhydrate the wounds, which may often cause infection and hamper wound healing. Thus, the equilibrium of water content, water absorption, permeability, water vapor transmission rate, and other characteristics are important requirements of the ideal wound dressing [57].

Also, considering the hybrids formed by bi-component systems, the smaller value of WVT was measured for the blends with CMC-250:PVA that presented higher miscibility compared to CMC-700:PVA hydrogels, based on the SD assays.

#### 2.3.6. Biological Tests—MTT Assays of Hydrogels

**Figure 14:**
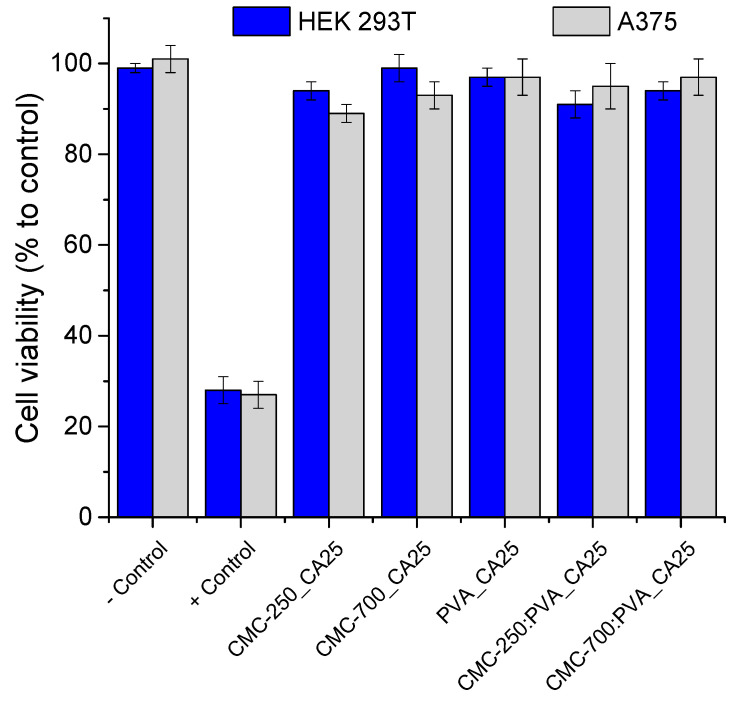
Cell viability results of HEK293T and A375 cell cultures based on MTT protocols after 24 h of incubation with hydrogels.

The cytocompatibility of the hydrogel membranes was preliminarily characterized in vitro through MTT assays to validate their potential to be applied in the biomedical field as wound dressings and skin repair materials [58,59,60]. Human embryonic cells (HEK293T) were used as a model line because they share similarities with epidermal skin cells (e.g., keratinocytes, dermal fibroblasts) that are involved in wound healing and soft tissue repair [58,59,60]. Human malignant melanoma (A375) was also tested. Figure 14 shows that hydrogels produced with pure polymers (CMC and PVA) and hybrids crosslinked with citric acid (CA/polymer = 25%) presented excellent cell viability responses of over 90%. These results demonstrated the non-toxicity of these materials, which are of pivotal importance for applications such as wound dressings and soft tissue repair (ISO 10993-5:2009, Biological evaluation of medical devices—Part 5: Tests for in vitro cytotoxicity).

## 3. Conclusions

Thus, this study reports the design, synthesis, and extensive characterization of hybrid hydrogels based on carboxymethyl cellulose and poly(vinyl alcohol) using citric acid as the eco-friendly chemical crosslinking agent, with bioengineered physicochemical properties and key characteristics appropriate to be applied as a wound dressing and skin repair tissue engineering. These polymeric hybrid matrices were produced through an entirely *green* process under mild conditions, where their properties were achieved using CMC biopolymers with two molecular weights, medium (250 kDa) and high (700 kDa), citric acid as chemical crosslinker at increasing concentrations (mass ratio) ranging from 0% (non-crosslinked) to 25%, and adding 20% PVA polymer (fully hydrolyzed, DH > 99%, Mw = 84–150 kDa) as the key network modifier. The results demonstrated that highly absorbent hydrogels were produced with a swelling degree spanning from 100% to 5000%, and a gel fraction from 40% to 80%. The concentration of CA crosslinker and the presence of PVA polymer as the CMC-based hybrid network modifier expressively governed the water-responsive behavior. Through in-depth and integrated analyses, the FTIR and XPS spectroscopic characterizations associated with thermal analysis demonstrated that the chemical crosslinking mechanism was predominantly related to the esterification chemical reaction between carboxylic groups from CA with hydroxyl groups of CMC and PVA macromolecules forming ester covalent bonds, rendering a crosslinked hybrid polymeric network. Moreover, these hybrid hydrogels presented hydrophilicity, permeability, and structural features dependent on the degree of crosslinking and relative composition of polymers (CMC and PVA) and CA, which were considered primarily suitable for wound dressing applications. More importantly, regarding the biological and biomedical applications, the hydrogels were cytocompatible considering the in vitro cell viability responses of over 90% towards human embryonic kidney cells (HEK293T) and human skin cancer cells (A375, human malignant melanoma) used as model cell lines. Hence, it may be affirmed that, based on the results presented above, the key strategy adopted in this study was effective, where hybrid hydrogels with adjustable physicochemical properties according to the projected specificities of the wound dressing were achieved. Furthermore, it can be anticipated that this research provides a groundwork for producing on-demand biocompatible hybrid hydrogels made of affordable and commercially available polymers with tailored properties for innovative wound dressings, which can be applied as temporary skin substitutes for assisting chronic wound healing and skin tissue engineering applications.

## 4. Materials and Methods

### 4.1. Materials

Sodium carboxymethyl cellulose salts with the degree of substitution DS = 0.81 ± 0.05, and two molar masses, Mw = 250 kDa (CMC-250) and Mw = 700 kDa (CMC-700), were purchased from Sigma-Aldrich (St. Louis, MO, USA). Poly(vinyl alcohol) (Mw = 85–124 kDa, degree of hydrolysis 99.3–100%) and citric acid (≥99.5%) were supplied by Sigma-Aldrich (USA). All polymers were used as received without further purification. Unless specified otherwise, DI-water (Simplicity® Water Purification System, Merck Millipore, Burlington, MA, USA) with a resistivity of 18 MΩ·cm was used to prepare the solutions, and the procedures were performed at room temperature (RT, 23 ± 2 °C). 

### 4.2. Synthesis of Chemically Crosslinked Polymer Hydrogels

A solution of CMC-250 (2% *w*/*v*) was prepared by adding polymer powder (4.0 g) to 200 mL of DI-water and stirring at room temperature until complete solubilization. For the CMC-700 solution using the same proportion (2% *w*/*v*), the temperature was raised to 60 ± 2 °C and stirred until complete solubilization. 

PVA was supplied as a powder, and the aqueous solution (2% *w*/*v*) was prepared by dissolving 4.0 g of polymer in 200 mL of DI-water. Firstly, PVA was dispersed in water at RT using sufficient magnetic stirring to wet out all particles. After five minutes, the temperature was increased to 87 ± 2 °C, and the magnetic stirring was reduced until the full dissolution of PVA. Polymer solutions were cooled down to room temperature before use when heating was used for solubilization. 

After the dissolution of polymers, the crosslinking agent, citric acid, was added under stirring at concentrations of CA/polymer (*w*/*w*) of 10% (CA10), 15% (CA15), 20% (CA20), and 25% (CA25) and homogenized for 20 min. In the sequence, 10 mL of the solutions were poured into plastic molds (polystyrene petri dish, 60 mm in diameter) and were allowed to dry at 40 ± 2 °C for 24 h for water removal, followed by thermal treatment at 80 ± 2 °C for 24 h for the crosslinking reaction. As the control sample, non-crosslinked hydrogels (without CA, termed CA0) were prepared following the same synthesis steps. 

### 4.3. Synthesis of Crosslinked CMC:PVA Hybrid Hydrogels

Solutions of CMC-250, CMC-700, and PVA were prepared as described in Section 4.2. Then, CMC:PVA mixtures were obtained by adding 20 mL of PVA solution to 80 mL of CMC solutions and mixing under magnetic stirring. After 30 min of homogenization, under stirring, the CA was added at concentrations of CA/(CMC + PVA) (*w*/*w*) of 15% (CMC:PVA_CA15), 20% (CMC:PVA_CA20), and 25% (CMC:PVA_CA25) and homogenized for 20 min. Afterward, the solutions were cast in plastic molds, dried, and thermally treated as described in Section 4.2. 

### 4.4. Characterization of Hydrogels

#### 4.4.1. Swelling Degree and Gel Fraction Tests

For swelling degree measurement and gel fraction assessments, the hydrogels were cut into 10 mm × 10 mm samples, dried at 40 ± 2 °C for mass stabilization, and weighted (W_0_, initial mass). Then, the hydrogels (replicates, n ≥ 3) were placed in 10.0 mL DI-water at RT. After 24 h, the hydrogel was removed from the solution, gently wiped to remove excess liquid on the sample surface, and weighed (W_s_, swollen mass). In the sequence, samples were dried at 40 ± 2 °C until mass stabilization, and the final weight was recorded (W_f_, final mass). 

The weight measurements obtained in each step of the process were used to calculate the hydrogels’ swelling degree and gel fraction using Equations (1) and (2), respectively, as reported in the literature [6,16,30,31,61]. Each measurement was performed in triplicate for both the swelling degree and solubility tests, and the results are expressed as the average ± standard deviation.

SD (%) = ((W_s_ − W_0_)/W_0_) × 100% (1)

GF (%) = ((W_0_ − W_f_)/W_0_) × 100% (2)

#### 4.4.2. Spectroscopic, Morphological, and Thermal Analyses

FTIR spectroscopy analysis was recorded with a Nicolet 6700 (Thermo Fisher Scientific Inc., Waltham, MA, USA) spectrometer with background subtraction. The FTIR spectra of hydrogel films were obtained using attenuated total reflectance (ATR) in the wavenumber range of 4000–750 cm^−1^, 32 scans, and a 4 cm^−1^ resolution (n ≥ 2).

The XPS analysis of hydrogels was performed using Mg-Kα as the excitation source at 120 W (Amicus spectrometer, Kratos, Kanagawa, Japan). All peak positions were corrected based on C 1s binding energy (285 eV). 

Thermogravimetric and differential scanning calorimetry analyses were performed using the SDT Q-600 instrument (TA Instruments Co., New Castle, Delaware). Hydrogel films of 1.5 ± 0.3 mg were tested at a heating rate of 10 °C.min^−1^ from RT to 500 °C. The samples were loaded into an open platinum pan, and an empty cup was used as a reference. The thermal analyses were performed under the continuous flow of dry nitrogen gas (30 mL.min^−1^) (n ≥ 2).

Scanning electron microscopy images were taken from the surface of hydrogels using a FEI–Inspect S50 microscope (FEI Company, Hillsboro, OR, USA). Before analysis, samples were coated with a thin gold film by sputtering, using a low deposition rate to avoid sample damage. 

#### 4.4.3. Thickness, Wettability, and Permeability Tests

The thickness of hydrogels was evaluated using a digital micrometer (Mitutoyo, Kanagawa, Japan). The results were based on five measurements, and the results are expressed as the average ± standard deviation.

The wettability of the hydrogels was evaluated by measuring the contact angle (ϕ) formed between the DI-water and the hydrogels. The hydrogel was assayed “as obtained” (“dry state”), similar to what was reported in several papers [54,62,63]. One droplet of DI-water was deposited onto the surface of each hydrogel using a microsyringe at RT, and digital images were acquired immediately after deposition (<5 s). The results represent the average angle between the tangent line at droplets and the surface of the hydrogel membranes (n ≥ 3).

The permeability to water vapor was determined by measuring the water vapor transmission using an adaptation of the ASTM E96/E96M-16 (Standard Test Method for Water Vapor Transmission of Materials). One sample of each hydrogel membrane was firmly attached to the top of a plastic dish containing 2.0 mL of distilled water and weighed (W_i_). The vials were then placed under controlled room conditions (temperature = 19 ± 2 °C, relative humidity of 50 ± 5%), and the dish assembly was weighed after 24 h (W_24h_). The water vapor transmission after 24 h (WVT) was determined according to Equation (3). The tests were performed in duplicate, and the results are expressed as the average.

WVT = (W_24h_ − W_i_)/(A × T) (3)

where A is the *cup mouth area* (7.5 cm^2^), and T is the time exposed to controlled climatic conditions (24 h, 1 day). 

#### 4.4.4. Cytotoxicity Tests

Human embryonic kidney cells (HEK293T, American Type Culture Collection—ATCC^®^ CRL-1573™) were provided by the Federal University of Minas Gerais (UFMG). Human malignant melanoma cells (A375, ATCC^®^ CRL-1619™, American Type Culture Collection, Rockville, MD, USA.) were purchased from Brazilian Cell Repository (Banco de Células do Rio de Janeiro: BCRJ, Rio de Janeiro, Brazil). All biological tests were conducted according to ISO 10993-5:2009/(R)2014 (biological evaluation of medical devices: tests for in vitro cytotoxicity). Before the experiments, the samples were sterilized by UV radiation for 60 min. 

The cytotoxicity of the hydrogels was evaluated using the directed contact 3-(4,5- dimethyl-2-thiazolyl)-2,5-diphenyltetrazolium bromide (MTT) method, as previously described by our group [64]. Both cells were cultured and tested in a Dulbecco’s Modified Eagle Medium (DMEM, pH 7.4 ± 0.2), and cells were exposed to 24 h of contact with samples. Percentage cell viability was calculated according to Equation (4). The values of the controls (wells with cells, and medium without samples) were set to 100% cell viability.

Cell viability (%) = (Absorbance of sample and cells/Absorbance of control) × 100% (4)

## Data Availability

All relevant data is available in the manuscript or the supporting information.

## Supplementary Materials

The following supporting information can be downloaded at: https://www.mdpi.com/article/10.3390/gels9020166/s1, Figure S1. FTIR spectra in the range of (A) 1850–1500 cm^−1^ and (B) 1300–1170 cm^−1^ of (a) CMC-700_CA0, (b) CMC-700_CA25 as synthesized, and (c) CMC-700_CA25 at pH 5.5. Figure S2. TG (a and b curves) and DTG (c and d curves) analysis of CMC-250_CA0 (—) and CMC-700_CA20 (**—**) hydrogels. Figure S3. Evolution of (A) OH---OH (3260 cm^−1^) with increasing CA content (inset: A_OH---OH_/A_CH3_). (B) Evolution of crystallinity of PVA with increasing citric acid content (inset: A_1141_/A_1096_).
