# Supplementary material for: Bioengineered Water-Responsive Carboxymethyl Cellulose/Poly(vinyl alcohol) Hydrogel Hybrids for Wound Dressing and Skin Tissue Engineering Applications
Source: Gels. 2023 Feb 18;9(2):166. doi: 10.3390/gels9020166 (PMC9956280; doi:10.3390/gels9020166)
Supplement: Supplementary file 1 [file gels-09-00166-s001.zip › gels-2212333-supplementary.pdf]

Supplementary Materials

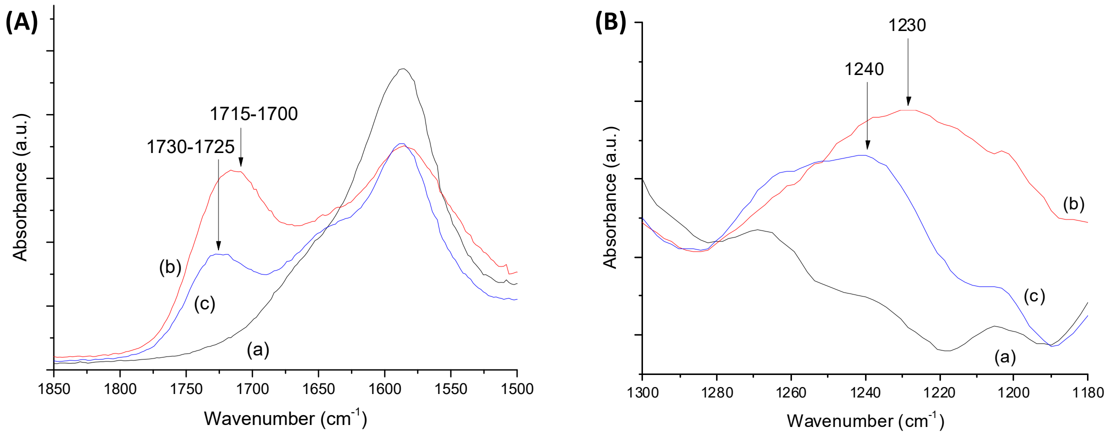

Figure S1. FTIR spectra in the range of (**A**) 1850–1500 cm^{-1} and (**B**) 1300–1170 cm^{-1} of (a) CMC-700_CA0, (b) CMC-700_CA25 as synthesized, and (c) CMC-700_CA25 at pH 5.5.

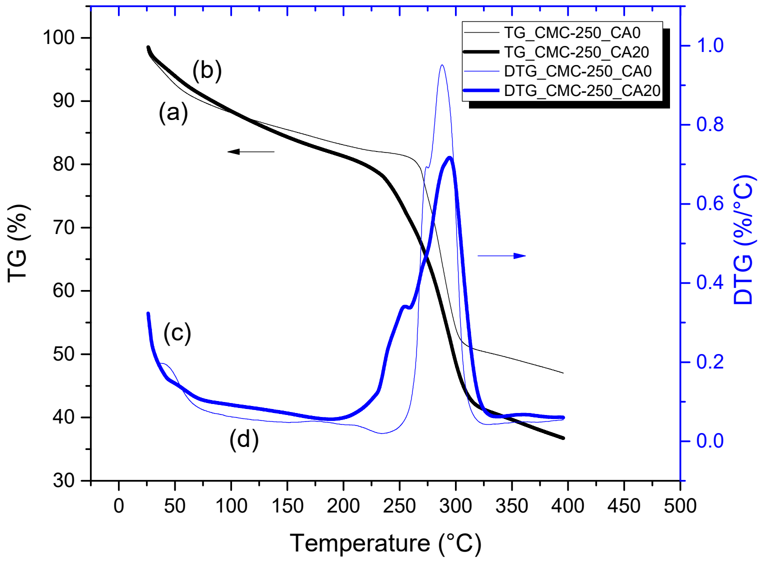

Figure S2. TG (a and b curves) and DTG (c and d curves) analysis of CMC-250_CA0 (—) and CMC-700_CA20 (—) hydrogels.

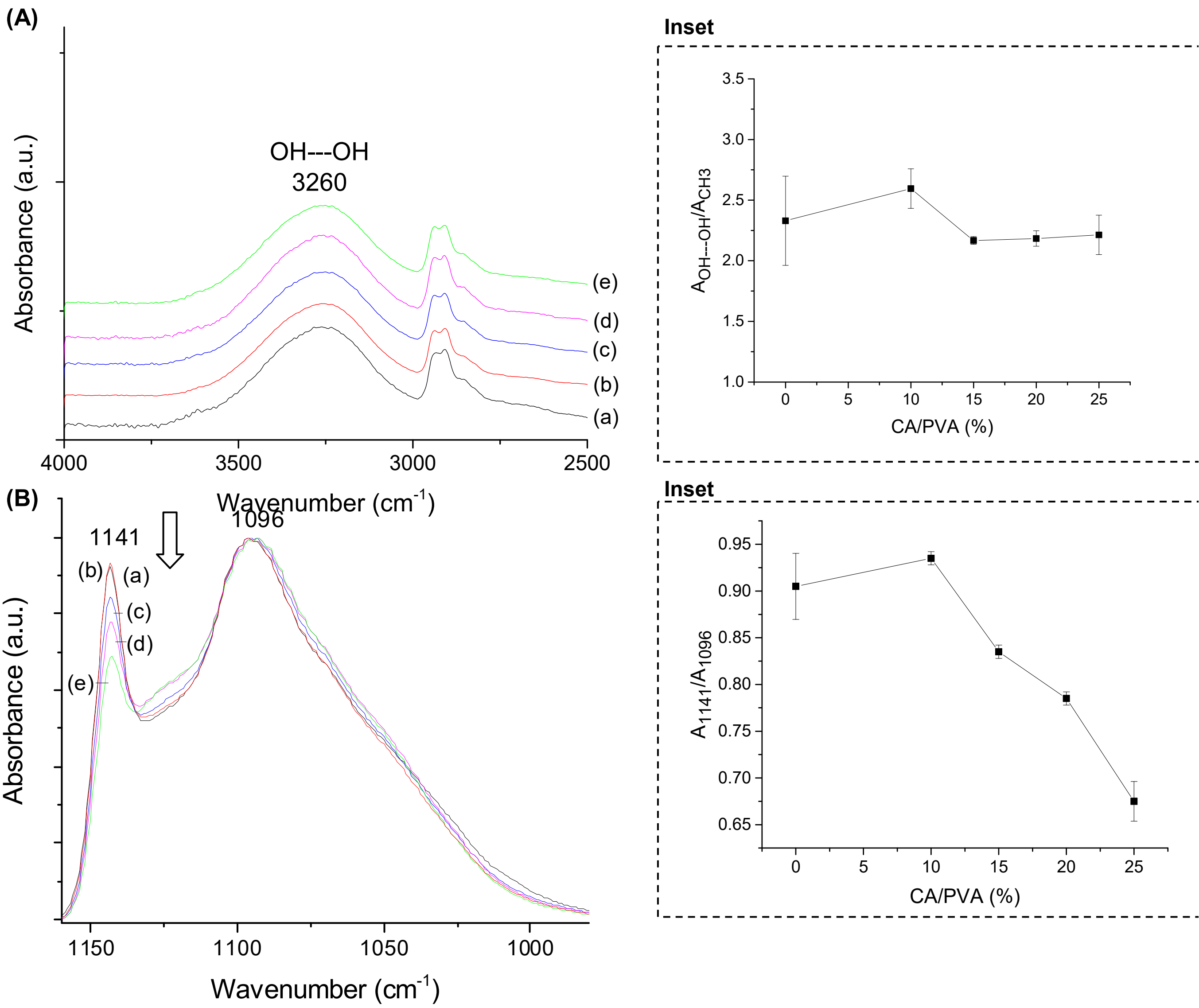

Figure S3. Evolution of (**A**) OH---OH (3260 cm^{-1}) with increasing CA content (inset: $A_{OH---OH}/A_{CH3}$). (**B**) Evolution of crystallinity of PVA with increasing citric acid content (inset: A_{1141}/A_{1096}).
